# Ectopic Expression of *Ptf1a* Induces Spinal Defects, Urogenital Defects, and Anorectal Malformations in *Danforth's Short Tail* Mice

**DOI:** 10.1371/journal.pgen.1003204

**Published:** 2013-02-21

**Authors:** Kei Semba, Kimi Araki, Ken-ichirou Matsumoto, Hiroko Suda, Takashi Ando, Akira Sei, Hiroshi Mizuta, Katsumasa Takagi, Mai Nakahara, Mayumi Muta, Gen Yamada, Naomi Nakagata, Aritoshi Iida, Shiro Ikegawa, Yusuke Nakamura, Masatake Araki, Kuniya Abe, Ken-ichi Yamamura

**Affiliations:** 1 Division of Developmental Genetics, Institute of Resource Development and Analysis, Kumamoto University, Kumamoto, Japan; 2 Department of Orthopedic and Neuro-Musculoskeletal Surgery, Faculty of Life Sciences, Kumamoto University, Kumamoto, Japan; 3 Department of Transplantation/Pediatric Surgery, Faculty of Life Sciences, Kumamoto University, Kumamoto, Japan; 4 Department of Organ Formation, Institute of Resource Development and Analysis, Kumamoto University, Kumamoto, Japan; 5 Division of Reproductive Engineering, Institute of Resource Development and Analysis, Kumamoto University, Kumamoto, Japan; 6 Laboratory for Bone and Joint Diseases, RIKEN Center for Genomic Medicine, Tokyo, Japan; 7 Human Genome Center, Institute of Medical Science, The University of Tokyo, Tokyo, Japan; 8 Division of Bioinformatics, Institute of Resource Development and Analysis, Kumamoto University, Kumamoto, Japan; 9 Technology Development Team for Mammalian Cellular Dynamics, BioResource Center, RIKEN, Tsukuba, Ibaraki, Japan; The Jackson Laboratory, United States of America

## Abstract

*Danforth's short tail* (*Sd*) is a semidominant mutation on mouse chromosome 2, characterized by spinal defects, urogenital defects, and anorectal malformations. However, the gene responsible for the *Sd* phenotype was unknown. In this study, we identified the molecular basis of the *Sd* mutation. By positional cloning, we identified the insertion of an early transposon in the *Sd* candidate locus approximately 12-kb upstream of *Ptf1a*. We found that insertion of the transposon caused overexpression of three neighboring genes, *Gm13344*, *Gm13336*, and *Ptf1a*, in *Sd* mutant embryos and that the *Sd* phenotype was not caused by disruption of an as-yet-unknown gene in the candidate locus. Using multiple knockout and knock-in mouse models, we demonstrated that misexpression of *Ptf1a*, but not of *Gm13344* or *Gm13336*, in the notochord, hindgut, cloaca, and mesonephros was sufficient to replicate the *Sd* phenotype. The ectopic expression of *Ptf1a* in the caudal embryo resulted in attenuated expression of *Cdx2* and its downstream target genes *T*, *Wnt3a*, and *Cyp26a1*; we conclude that this is the molecular basis of the *Sd* phenotype. Analysis of *Sd* mutant mice will provide insight into the development of the spinal column, anus, and kidney.

## Author Summary

Caudal regression syndrome (CRS) is a congenital heterogeneous constellation of caudal anomalies that includes varying degrees of agenesis of the spinal column, anorectal malformations, and genitourinary anomalies. Its pathogenesis is unclear. However, it could be the result of excessive physiologic regression of the embryonic caudal region based on analyses of the various mouse mutants carrying caudal agenesis. Among the mouse mutants, the *Danforth's short tail* (*Sd*) mouse is considered a best model for human CRS. *Sd* is a semidominant mutation, characterized by spinal defects, urogenital defects, and anorectal malformations, thus showing phenotypic similarity to human CRS. Although *Sd* is known to map to mouse chromosome 2, little is known about the molecular nature of the mutation. Here, we demonstrate an insertion of one type of retrotransposon near the *Ptf1a* gene. This resulted in ectopic expression of *Ptf1a* gene in the caudal region of the embryo and downregulation of *Cdx2* and its downstream targets, leading to characteristic phenotypes in *Sd* mouse. Thus, *Sd* mutant mice will provide insight into the development of the spinal column, anus, and kidney.

## Introduction

*Danforth's short tail* (*Sd*) is a semidominant spontaneous mutant mouse characterized by severe spinal defects, urogenital defects, and anorectal malformations [1], [2], [3]. Heterozygous and homozygous *Sd* animals display a broad range of abnormalities in the vertebral column, including reduction or absence of the dens axis, reduction of all vertebral bodies in the dorsoventral axis, split vertebrae, and truncation of the caudal vertebral column [4], [5], [6]. The vertebral columns of *Sd/Sd* and *Sd/+* mice are usually truncated at the seventh thoracic and the sixth caudal vertebral body, respectively [7]. The urogenital system in *Sd* heterozygotes may display malformations ranging from displaced to missing kidneys. Homozygotes invariably have missing or severely malformed and dislocated kidneys. The rectum and anal opening are missing, and the embryonic cloaca persists. Homozygous animals die within 24 h after birth [4].

Although *Sd* is known to map to mouse chromosome 2, little is known about the molecular nature of the mutation. Double mutants between the *Sd* and undulated (*un*) alleles showed reduced expression of *Pax1* and enhancement of the vertebral malformations [8]. *Pax1* expression is regulated by signals from the notochord [9], [10], thus providing a potential molecular link for the interaction between *un* and *Sd*. Zachgo et al. obtained a *lacZ* enhancer trap insertion called *Etl4^lacZ^*, which is tightly linked to *Sd*. If *Etl4^lacZ^* is present in trans (i.e., on the chromosome that is wild type (WT) for *Sd*), the *Sd* phenotype is enhanced [11]. In contrast, if *Etl4^lacZ^* is present in *cis* (i.e., on the same chromosome as *Sd*), the phenotype is attenuated, suggesting a direct interaction of the transgene insertion with the *Sd* gene at the DNA level. However, neither the *Sd* mutation nor the *Sd* gene is known [12], [13].

We previously obtained a mutant mouse line, *Skt^Gt^*, through gene-trap mutagenesis, and identified the *Skt* gene. We found that the *Skt^Gt^* locus was located 0.95 cM distal to the *Sd* locus, and that the *Etl4^lacZ^* site was located in the third intron of the *Skt* gene [14]. Because the *Sd* region had been shown to be located 0.15–0.3 cM distal to the marker *D2Mit362* [13], [15] and 0.75 cM proximal to *Etl4^lacZ^* [11], it was clear that the *Sd* locus is located between *D2Mit362* and the *Skt* gene. In this study, we identified the cause of the *Sd* mutation by generating an *Sd*-derived cosmid contig of approximately 0.5-Mb around the *Sd* locus and using it to guide the production of genetically engineered mice with particular genetic alterations.

## Results

### Phenotype of *Sd* mice

**Figure 1:**
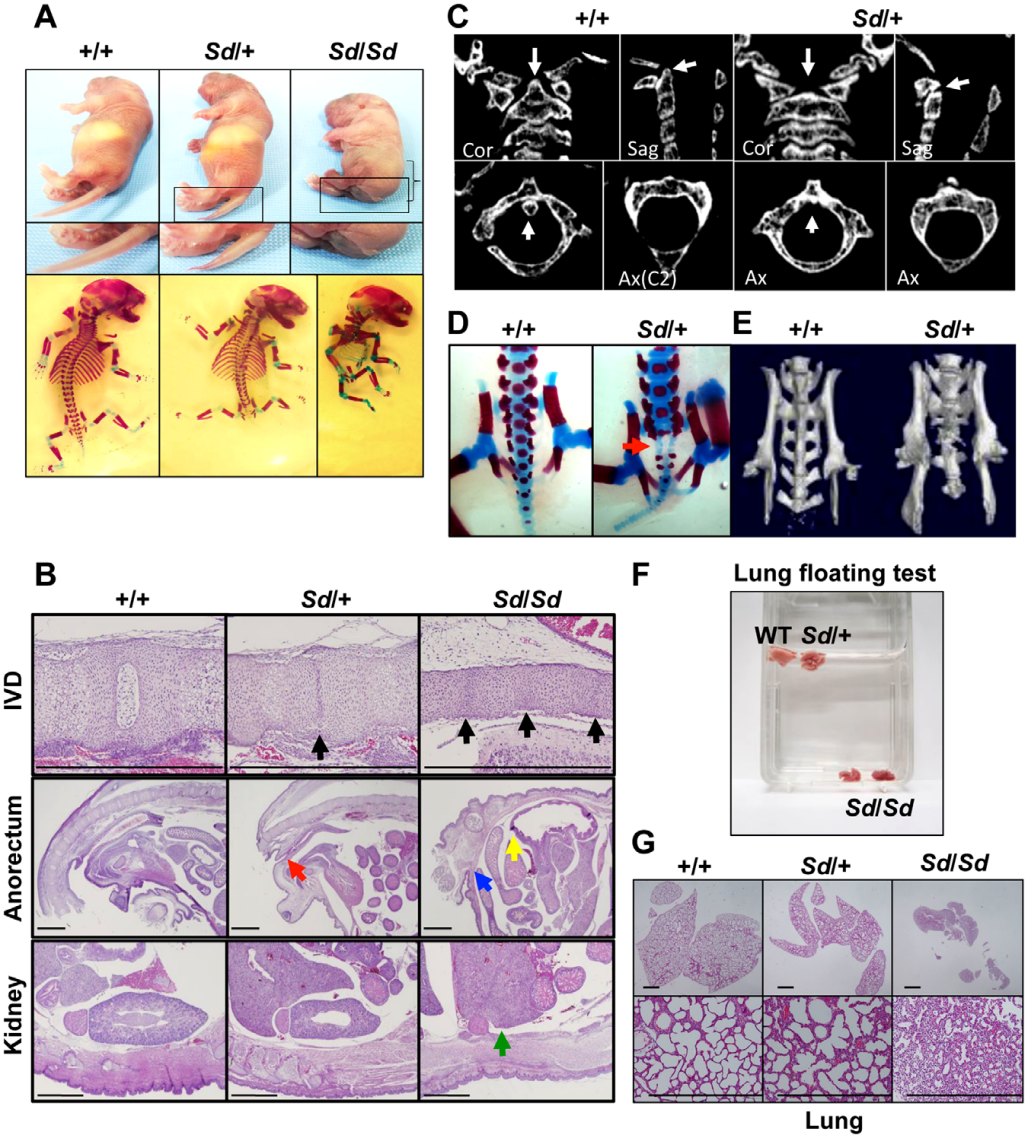
Morphology of *Sd* mice. A. Truncation of tail and axial skeletal defects at day 0 postpartum. *Sd* homozygotes have similar, but much more severe, abnormalities than heterozygotes. The bracket indicates a short body trunk. B. Hematoxylin and eosin-stained histological sections of the thoracic intervertebral discs (IVDs), anorectum, and kidney of E18.5 embryos. Defects of the nucleus pulposus of the IVDs (black arrows) in hypoplastic intervertebral bodies were observed in both *Sd*/+ and *Sd*/*Sd* embryos. Sagittal sections of the anorectum showed blind-end-type anorectal malformations in *Sd*/*Sd* mice (yellow and blue arrows), and anal stenosis in *Sd*/+ (red arrow) mice. Sagittal sections of the kidney revealed renal agenesis (green arrow) in *Sd*/*Sd* mice. Bars: 1 mm. C. High-resolution computed tomography images of the cervical vertebral bodies. Upper panel: Coronal (Cor) and sagittal (Sag) images. Lower panel: Axial (Ax) images. The arrows in the wild-type (+/+) mice indicate the position of the dens in the cervical vertebral bodies. *Sd*/*+* mice showed hypoplasia of the dens, as indicated by arrows. D. Alizarin red S and alcian blue skeletal staining of E18.5 embryos. *Sd/+* embryos showed partial sacral defects that developed before birth (red arrow). E. High-resolution three-dimensional computed tomography images. *Sd/+* mutant mice showed sacral hypoplasia, similar to that of Currarino syndrome. F. Lungs from *Sd*/*Sd*, but not wild-type (+/+) or *Sd*/+ mice, sank in water. G. Hematoxylin and eosin-stained histological sections showed atelectasis of *Sd/Sd* neonates' lungs, indicating no sign of breathing after birth. Bars: 500 µm.

Although the *Sd* mouse was identified in 1940, detailed histological findings have not yet been fully described. In this study, we mainly analyzed three tissues—the vertebrae, urogenital tract, and kidney—because characteristic features are found in these tissues of *Sd* mice. *Sd*/*Sd* homozygotes had similar, but much more severe, abnormalities than *Sd*/*+* heterozygotes in terms of truncation of the vertebrae at day 0 postpartum (Figure 1A, Text S1), defects of the nucleus pulposus in the intervertebral discs, anorectal malformations, and renal hypoplasia/agenesis (Figure 1B). We also revealed hypoplasia of the dens (Figure 1C) and sacral hypoplasia (Figure 1D, 1E) by high-resolution computed tomography (Text S1). Thus, *Sd* is considered a mouse model for caudal regression syndrome (CRS), characterized by vertebral, anorectal, and urogenital abnormalities. We also carried out a lung-floating test to analyze the cause of death. As shown in Figure 1F, the lungs of *Sd/Sd* neonates sank in water. Histological sections of the lung revealed atelectasis of the lung (Figure 1G), indicating that no breathing occurred after birth.

### Insertion of a retrotransposon in the *Sd* mouse genome

The *Sd* locus had been shown to be located 0.15–0.3 cM distal to *D2Mit362* and 0.75 cM proximal to *Etl4^lacZ^* [11], [13], [15]. We demonstrated that the insertion site of *lac*Z in the *Etl4^lacZ^* mutation was within the third intron of *Skt* [14]; thus, it was clear that *Sd* is located between *D2Mit362* and *Skt*. Furthermore, we showed that the genetic distance between *Skt^Gt^* and *Sd* was 0.95 cM, and hence that *Skt* was genetically separated from *Sd* [14].

We created a cosmid library using embryonic day (E) 11.5 homozygous *Sd/Sd* embryos. We screened this cosmid library with 32 different DNA probes, obtaining 28 cosmid clones within the *Sd* region between *D2Mit362* and *Skt^Gt^*. Based on physical mapping of 19 cosmid clones and 25 PCR products, the assembled contig spans a 542-kb region containing *Sd* (Figure S1). The region spanned by the contig contained three known genes, *Ptf1a*, *Msrb2*, and *Skt*, and five expressed sequence tags of unknown function: *Gm13344*, *Gm13336*, *4921504*, *E06Rik*, and *Otud1* (Figure S1, Text S1).

**Figure 2:**
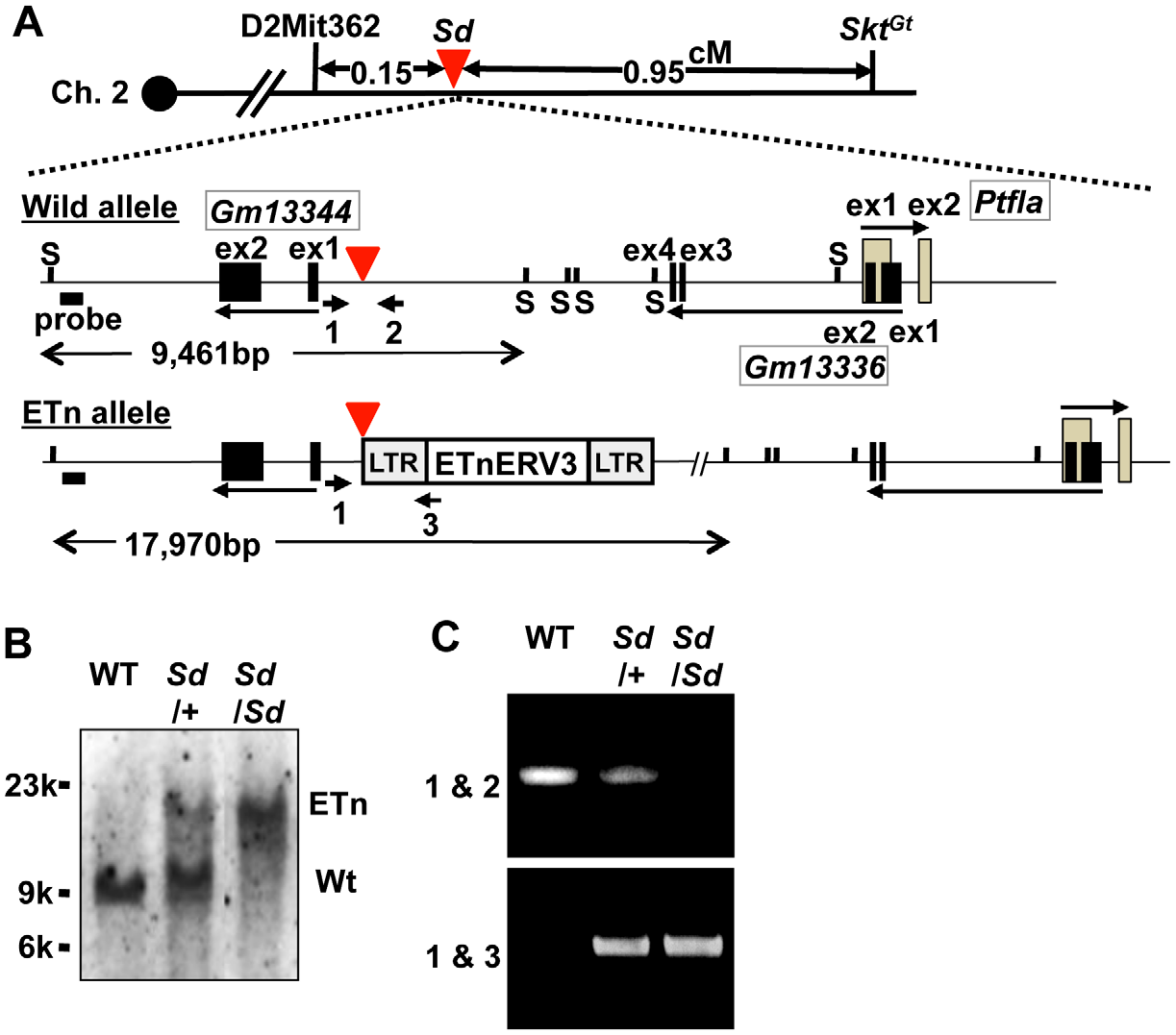
Insertion of a retrotransposon in the *Sd* mouse. A. Structure of the wild-type and ETn alleles. The 8,497-bp of ETnERV3 was inserted 1,418-bp upstream of the first exon of *Gm13344* and 8,114-bp downstream of the last exon of *Gm13336*. Each box indicates an exon. Arrows indicate the direction of transcription of the *Gm13344*, *Gm13336*, and *Ptf1a* genes. Black bold bars indicate genomic probes used for Southern blotting. “S” indicates Sph I restriction sites. Bold arrows with numbers indicate primers used for PCR analyses. The red arrowhead indicates the insertion point of the ETn. B. Genotyping by Southern blotting. An approximately 9.4-kb band or 18-kb band corresponded to the wild-type (WT) and ETn alleles, respectively. C. Genotyping by genomic PCR analysis. Primer pairs 1 and 2 or 1 and 3 were used to detect the WT or ETn alleles, respectively.

Interestingly, we found one cosmid clone for which one Not I digestion product was longer than that expected based on its end-sequence tags and wild-type genome informatics (C57BL/6 and Sv-129). We performed shotgun sequencing of the insert of this cosmid and determined it to be 36,440-bp long, which was considerably longer than the expected 27,936-bp (Figure S2A–S2C, Text S1). We submitted the 36,440-bp sequence to GenBank with accession number AB70168. Sequence analysis revealed that this cosmid contained a retrotransposon near the *Gm13344*, *Gm13336*, and *Ptf1a* genes. This retrotransposon was highly homologous to murine early transposon (ETn) endogenous retrovirus (ERV) 3 (ETnERV3)—Family: ERVK, Class: long-terminal repeat (98.6% identity)—whose size was 8,497 nucleotides (AB701682) (Figure 2A). This ETn was found to be tightly linked to the *Sd* phenotype, showing no recombination in 1,157 offspring. We performed Southern blot analysis using flanking DNA-specific genomic probes from the 5′-region to genotype *Sd*/*Sd*, *Sd*/+, and WT (+/+) mice. One 17,970-bp and one 9,461-bp band were detected in *Sd*/*Sd* E18.5 and +/+ embryos, respectively. Both were detected in *Sd*/+ E18.5 embryos (Figure 2B). In addition, using PCR primer pairs shown in Figure 2A, the genotype of offspring from the heterozygous intercross could be easily determined (Figure 2C). These data clearly suggest that the ETn insertion is associated with *Sd* phenotypes.

### *Sd* phenotypes are caused by the ETn insertion

The ETn insertion may disrupt an as-yet-unknown gene present in the insertion site or the presence of the ETn itself may cause the *Sd* phenotype. To distinguish these possibilities, we used a method that we developed for exchangeable gene targeting using Cre/*lox* [16], [17], [18]. Using this system, we were able to disrupt the target locus in the first step, and then produce an ETn knock-in allele (kiETn) by Cre-mediated site-specific recombination.

**Figure 3:**
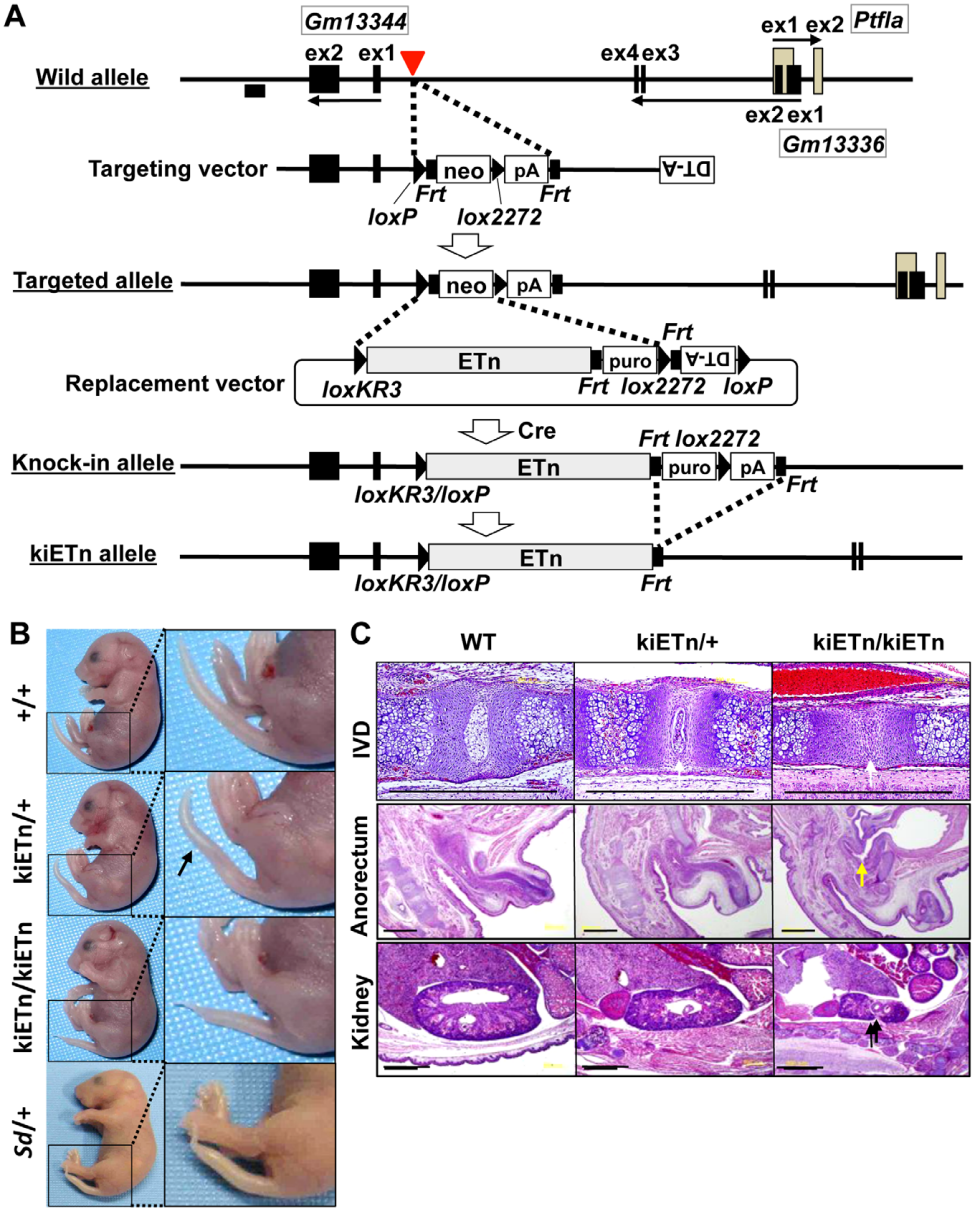
*Sd* phenotypes are caused by the ETn insertion. A. Strategy for creation of the knock-in (ki)ETn allele. The targeting vector was first used to create a targeted allele containing a neomycin resistance (*neo*) gene flanked by *loxP* and *lox2272*. Then, a replacement vector was used to replace *neo* with ETn by Cre-mediated recombination. Exons are shown as gray or black boxes. Black arrows indicate the direction of gene transcription. The red arrowhead indicates the insertion point of the ETn. The probe used for genotyping is shown as a black box. B. Morphology of the tails of E18.5 embryos. The kiETn/+ and the kiETn/kiETn embryos showed a kinked (arrow) and truncated tail, respectively. C. Hematoxylin and eosin staining of the intervertebral discs (IVDs), anorectum, and kidney of E18.5 embryos. The kiETn/+ embryos showed hypoplasia of the nucleus pulposus of the IVDs (white arrows). The kiETn/kiETn embryos showed a nucleus pulposus defect (white arrow) in the IVD, an anorectal malformation that was classified as fistulas of the urogenital tract (yellow arrow), and hypoplasia of the kidney (black arrow). Bars: 1 mm.

To examine whether the *Sd* phenotype was caused by disruption of an as-yet-unknown gene present at the insertion site of the ETn, we constructed a targeting vector that contained a 5′ homology region, the neomycin resistance gene (*neo*) flanked by *loxP* and *lox2272*, and a 3′ homology region. Using this targeting vector, we inserted *neo* flanked by *loxP* and *lox2272* into embryonic stem (ES) cells at the site where the ETn is found in the *Sd* mouse (Figure 3A). We obtained *neo* knock-in mice through mating of germline chimeras (Figure S3A, S3B). The *neo* mice did not show any phenotype (Figure S3C). Thus, we could rule out the possibility that *Sd* is caused by the disruption of an as-yet-unidentified gene at the insertion site of the ETn.

We then examined whether the presence of ETn is required for the *Sd* phenotype. We first isolated an Xba I fragment containing the ETn with 287-bp upstream and 630-bp downstream regions from cosmid clone C3. Then, we prepared a replacement vector that contained the Xba I fragment flanked by *loxKR3* and *lox2272*. By electroporating this replacement vector and a Cre expression vector, we created a knock-in allele in ES cells in which *neo* was replaced with ETn. The puromycin resistance (*puro*) gene was removed by expressing Flp, thus producing the kiETn allele (Figure 3A). The established kiETn heterozygous animals were fertile and showed a kinked tail phenotype. In this experiment, none of wild type mice (0/29) showed kinky tail, while 27% (6/22) kiETn/+ mice showed kinky tail (Figure 3B). There was no sex difference. Homozygotes showed characteristic *Sd* phenotypes, such as short tails with a defect of the nucleus pulposus of the intervertebral discs, anorectal malformations, and hypoplasia of the kidney, which were more severe than those in *Sd* heterozygotes (Figure 3B, 3C). All homozygous kiETn animals showed perinatal lethality. These data strongly supported the hypothesis that the ETn insertion is the cause of the *Sd* phenotype. However, the kiETn mutant phenotype was less severe than that observed in *Sd* mice. The kiETn allele was produced using an Xba I fragment containing the ETn with 287-bp 5′ and 630-bp 3′ flanking genomic sequences; these genomic flanking regions were duplicated in the kiETn allele. In addition, the *loxKR3/loxP* and *Frt* sequences were retained in the kiETn allele. Therefore, it is possible that the retention of these sequences attenuated the effect of the ETn on the phenotype.

### Increased expression of *Gm13344*, *Gm13336,* and *Ptf1a* transcripts

**Figure 4:**
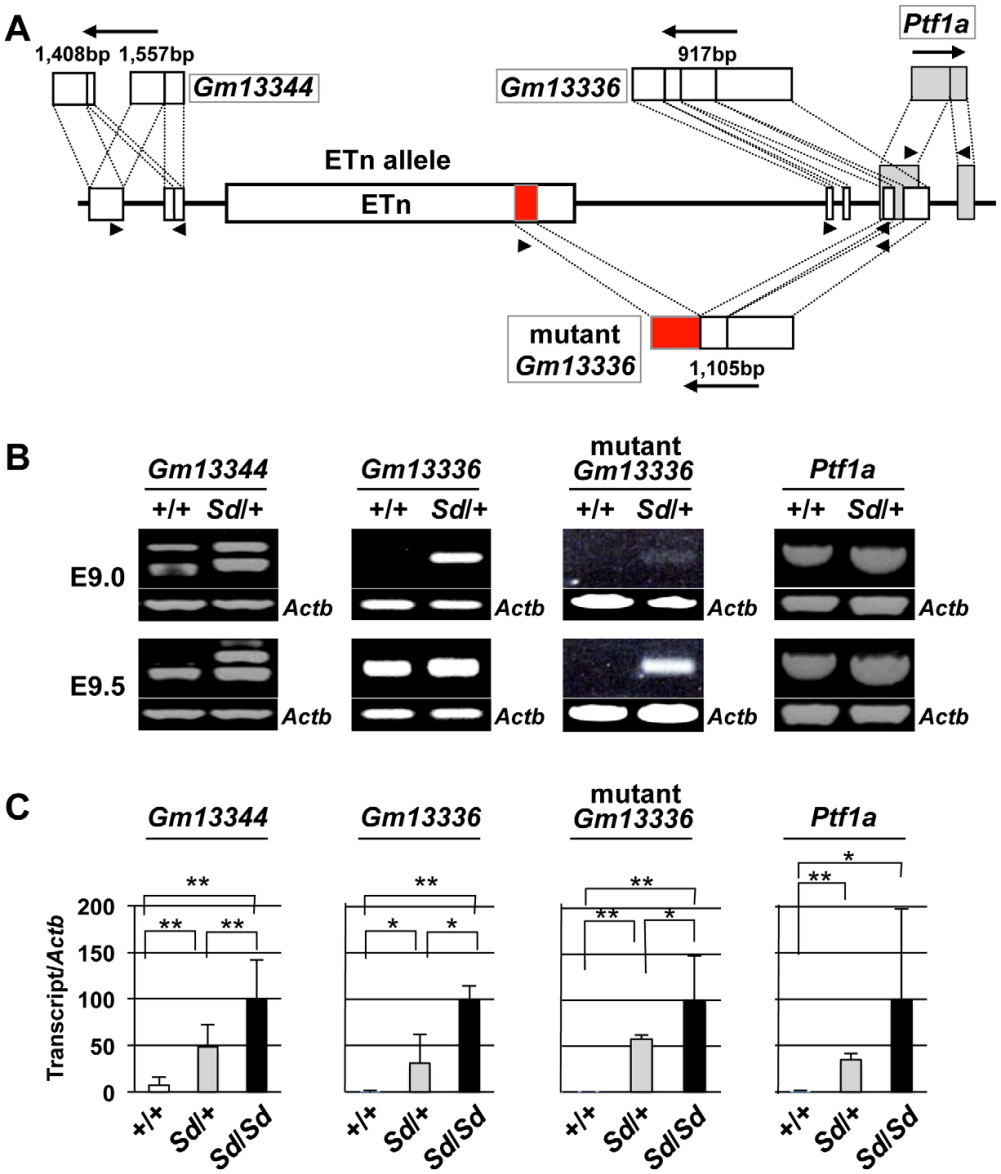
Increased expression of transcripts from the *Gm13344* and *Gm13336-Ptf1a* loci. A. Schematic representation of transcripts. Two alternatively spliced transcripts were produced from the *Gm13344* locus. One transcript was produced from each of the *Gm13336* locus and the *Ptf1a* locus. In addition, one fusion transcript was produced from *Gm13336* and the ETn. The part of the ETn sequence contained in the fusion transcript is shown as a red box. Each exon of *Gm13344*, *Gm13336*, and *Ptf1a* is shown as a white or gray box. The black arrows indicate the direction of transcription. Triangles indicate PCR primers. B. RT-PCR analyses. Increased expression of *Gm13344*, normal *Gm13336*, mutant *Gm13336*, and *Ptf1a* was observed in E9.0 and E9.5 *Sd/+* embryos. C. Quantitative RT-PCR analyses. Expression of *Gm13344*, *Gm13336*, mutant *Gm13336*, and *Ptf1a* was increased in E9.5 embryos of *Sd*/+ and *Sd*/*Sd* littermates. The data represent the mean ± SD of independent whole embryos (*Gm13344*, +/+: *n* = 4, *Sd*/+: *n* = 6, *Sd*/*Sd*: *n* = 6; *Gm13336*, +/+: *n* = 5, *Sd*/+: *n* = 5, *Sd*/*Sd*: *n* = 3; mutant *Gm13336*, +/+: *n* = 5, *Sd*/+: *n* = 5, *Sd*/*Sd*: *n* = 3; *Ptf1a*, +/+: *n* = 8, *Sd*/+: *n* = 16, *Sd*/*Sd*: *n* = 10). *p<0.05; ** *p*<0.01.

To examine the effects of the ETn on transcription of the *Gm13344*, *Gm13336*, and *Ptf1a* genes, we first obtained full-length sequences for the *Gm13344*, *Gm13336*, and *Ptf1a* transcripts by rapid amplification of cDNA ends (RACE) and reverse transcription PCR (RT-PCR) (Text S1). The full-length sequences of the *Gm13344*, *Gm13336*, and *Ptf1a* transcripts were determined by compiling the sequences of the 5′ RACE and 3′ RACE products with those of expressed sequence tags that showed 100% homology to the RACE products. The final *Gm13344* and *Gm13336* cDNA sequences were 1,557-bp (AB701678) and 917-bp (AB701680) long, respectively (Figure 4A). In the case of *Gm13344*, we identified two alternative splicing products, 1,557-bp and 1,403-bp (AB701679) long; splicing in the first exon produced the latter transcript. Unexpectedly, we found a 1,105-bp fusion transcript (AB701681) containing the first and second exons of *Gm13336* and 456-bp of the ETn sequence in *Sd* mutant embryos, which we termed the mutant *Gm13336* transcript (m*Gm13336*) (Figure 4A). Our sequencing data suggested that the *Gm13344* and *Gm13336* genes do not contain a significant open reading frame (ORF). Moreover, *Gm13344* partially overlaps with *Gm13336*, and *Gm13336* partially overlaps with *Ptf1a*. Interestingly, quantitative RT-PCR analyses revealed increased expression of all four transcripts in the *Sd* mutant at E9.0 and E9.5 (Figure 4B, 4C), but not at E10.0.

### Tail phenotype in ETn-*Gm13336*-*Ptf1a* transgenic mice

**Figure 5:**
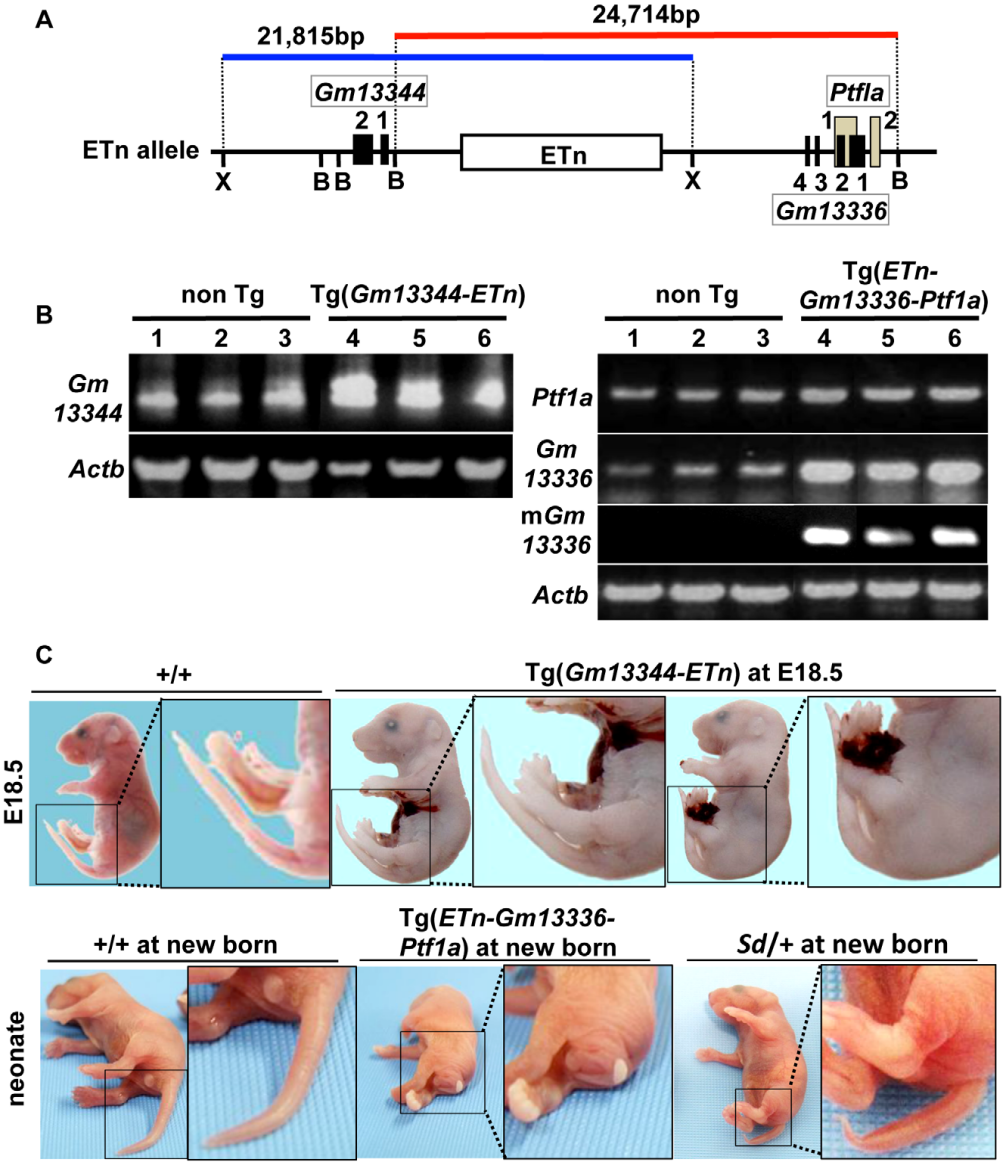
Tail phenotype in *Gm13336-Ptf1a* transgenic mice. A. Structure of the transgene containing the ETn. Schematic map of the ETn insertion locus. The proximal Xho I (X) fragment spanning 21,815-bp (blue bar) contains *Gm13344* and the ETn. The distal Bsm BI fragment spanning 24,714-bp (red bar) contains the ETn and *Gm13336-Ptf1a*. Exons of the *Gm13344*, *Gm13336*, and *Ptf1a* genes are shown as black or gray boxes. Numbers indicate corresponding exon numbers. B. RT-PCR analyses. Increased expression of *Gm13344* in transgenic (Tg)(ETn-*Gm13344*) and of *Gm13336*, m*Gm13336*, and *Ptf1a* in Tg(ETn-*Gm13336-Ptf1a*) embryos was observed at E10.5. C. Morphology of the tail in E18.5 embryos and neonates. Tg(*ETn-Gm13344*) E18.5 embryos showed a normal tail. Tg(*ETn-Gm13336-Ptf1a*) and *Sd/+* neonates showed short tails.

To determine whether any of these four transcripts is responsible for *Sd*, we first generated two lines of transgenic mice carrying either a proximal genomic fragment including *Gm13344* and the ETn (*Gm13344*-ETn) or a distal genomic fragment including the ETn, *Gm13336*, and *Ptf1a* (ETn-*Gm13336*-*Ptf1a*) (Figure 5A). Although all four transcripts were expressed in transgenic mice at E10.5 or neonatally (Figure 5B), *Gm13344*-ETn transgenic embryos showed no phenotype (Figure 5C), while ETn-*Gm13336*-*Ptf1a* transgenic mice showed a short tail similar to that of the *Sd* mutant (Figure 5C). These results strongly suggested that the increased expression of *Gm13336*, m*Gm13336*, and/or *Ptf1a* is the cause of *Sd*.

### Phenotypic rescue by disruption of the *Gm13336* and *Ptf1a* genes

**Figure 6:**
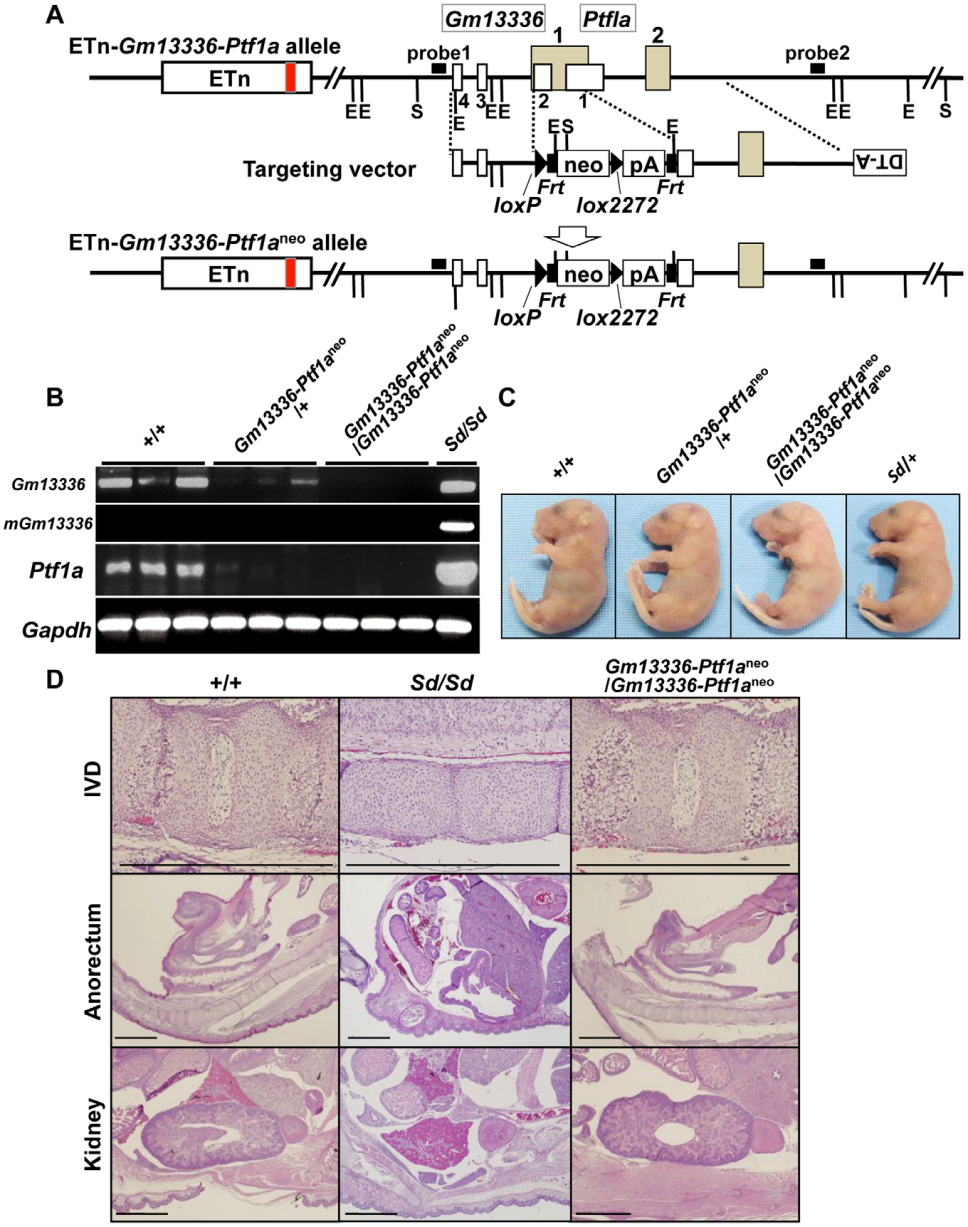
Phenotypic rescue by disruption of the *Gm13336-Ptf1a* gene. A. Strategy for disruption of the *Gm13336-Ptf1a* gene. The *Gm13336-Ptf1a* gene was disrupted using a targeting vector that contained a neomycin resistance (*neo*) gene flanked by *loxP* and *lox2272*. Exons of the *Gm13336* and *Ptf1a* genes are shown as white and gray boxes, respectively. B. RT–PCR analyses of E9.5 embryos. Expression of *Gm13336*, mutant *Gm13336*, and *Ptf1a* was not detected in ETn-*Gm13336-Ptf1a* ^neo^/ETn-*Gm13336-Ptf1a* ^neo^ embryos. C. Tail phenotype of E18.5 embryos. Both ETn-*Gm13336-Ptf1a* ^neo^/+-+ and ETn-*Gm13336-Ptf1a* ^neo^/ETn-*Gm13336-Ptf1a* ^neo^ embryos showed normal tails, although these embryos have the ETn. D. Hematoxylin and eosin-stained sections from E18.5 embryos. The *Sd* phenotype observed in the intervertebral discs (IVDs), anorectum, and kidneys was not present in ETn-*Gm13336-Ptf1a* ^neo^/ETn-*Gm13336-Ptf1a* ^neo^ mice. Bars: 1 mm.

Because *Gm13336* and *Ptf1a* overlap each other, the strategy we employed to identify the *Sd* gene was as follows. First, we created a null allele for all three transcripts—*Gm13336*, m*Gm13336*, and *Ptf1a*. Then, we expressed each gene by returning it into this locus by the exchangeable gene targeting method, to determine whether the *Sd* phenotype could be reproduced. For this purpose, we successfully obtained germline-competent ES cell lines using blastocysts obtained by mating *Sd*/+ mice and +/+ mice. Four of 15 ES cell lines carried the *Sd* allele and three were positive for the *Sry* gene (Figure S4A, Text S1). All the chimeric mice showed a short tail (Figure S4B). We used this germline-competent *Sd*/+ ES cell line for disruption of the *Gm13336*-*Ptf1a* allele. The vector used for homologous recombination in *Sd*/+ ES cells is shown in Figure 6A. The *neo* cassette was inserted between the first and second exons of *Gm13336*, resulting in deletion of the first exon of *Ptf1a*. Nine targeted ES clones lacking *Gm13336-Ptf1a* were identified by Southern blot analysis with both a 5′ probe and a 3′ probe (Figure 6A) and were used to generate chimeric mice. We obtained eight chimeric mice and four of them were germline chimeras. The *Gm13336-Ptf1a* ^+/neo^ mice were healthy and fertile, and indistinguishable from their negative littermates. Then, we examined whether the *Gm13336-Ptf1a* ^neo^ allele and the ETn segregated in the next generation. We obtained 20 offspring from three germline chimeras. None showed segregation of the *Gm13336-Ptf1a^neo^* and ETn alleles (Figure S5A), suggesting that these offspring carry the *Gm13336-Ptf1a^neo^* and ETn alleles on the same chromosome [ETn-*Gm13336*-*Ptf1a* ^neo^/+-+; *cis* configuration]. These mice were intercrossed to produce homozygous ETn-*Gm13336-Ptf1a* ^neo^/ETn-*Gm13336-Ptf1a* ^neo^ mice. The ratio of *+/+*, ETn-*Gm13336-Ptf1a* ^neo^/+-+, and ETn-*Gm13336-Ptf1a* ^neo^/ETn-*Gm13336-Ptf1a* ^neo^ mice at E18.5 was 13∶25∶15 (*n* = 53), which is accordance with the expected Mendelian ratio. In ETn-*Gm13336-Ptf1a* ^neo^/ETn-*Gm13336-Ptf1a* ^neo^ embryos, no *Gm13336* or *Ptf1a* transcripts were detected (Figure 6B), indicating the creation of a null allele. Interestingly, ETn-*Gm13336-Ptf1a* ^neo^/ETn-*Gm13336-Ptf1a* ^neo^ embryos exhibited no abnormalities in vertebral, urogenital, or anorectal development (Figure 6C, 6D), despite the presence of the ETn allele. However, the pancreas was missing in ETn-*Gm13336-Ptf1a* ^neo^/ETn-*Gm13336-Ptf1a* ^neo^ E18.5 embryos, as expected (Figure S5B). Using the targeted ES clone, we obtained one germline chimera, which transmitted the ETn and *Gm13336-Ptf1a* ^neo^ alleles independently into its offspring. In this case, F1 mice carried the ETn and the *Gm13336-Ptf1a* ^neo^ alleles on different chromosomes [ETn-+/+-*Gm13336*-*Ptf1a* ^neo^; *trans* configuration] and thus showed a similar phenotype to that of the heterozygous *Sd* mutant. These findings clearly indicated that the *cis* configuration of ETn-*Gm13336-Ptf1a* causes the *Sd* phenotype.

### Identification of *Ptf1a* as a cause of *Sd*

**Figure 7:**
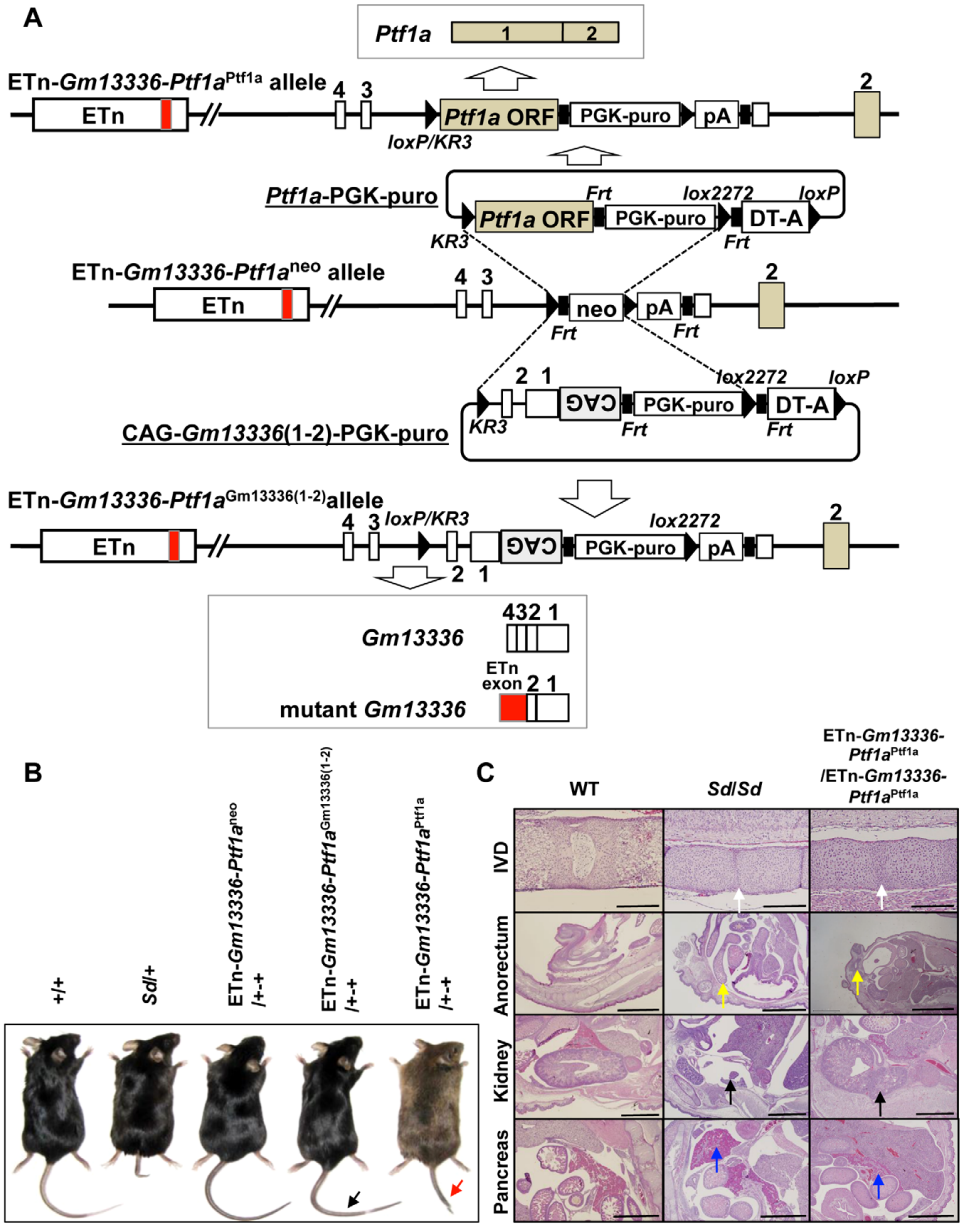
Identification of *Ptf1a* as a cause of *Sd.* A. Strategy for creation of a knock-in allele. Using the ES cell line carrying the ETn-*Gm13336-Ptf1a* ^neo^ allele, the neomycin resistance (*neo*) gene was replaced with the *Ptf1a* ORF or CAG-*Gm13336*(1–2) using the replacement vector *Ptf1a* ORF-PGK-*puro* or CAG-*Gm13336*(1–2)-PGK-*puro*, respectively. Exons are shown as white, gray or red boxes. B. Tail phenotype in adult mice. ETn-*Gm13336-Ptf1a* ^CAG-*Gm13336(1–2)*^/+-+ mice showed a normal tail (black arrow), despite the rescue of *Gm13336* and m*Gm13336* expression. However, ETn-*Gm13336-Ptf1a^Ptf1a^*/+-+ mice showed a short tail (red arrow). C. Hematoxylin and eosin-stained sections of neonates. Defects of the nucleus pulposus of the intervertebral discs (IVDs) (white arrows) and anorectal malformations (yellow arrows) in ETn-*Gm13336-Ptf1a* ^Ptf1a^/ETn-*Gm13336-Ptf1a* ^Ptf1a^ mice were similar to those in *Sd* homozygous mutants, although the kidney defect (black arrows) was less severe. The defect of the pancreas was restored to normal in ETn-*Gm13336-Ptf1a* ^Ptf1a^/ETn-*Gm13336-Ptf1a* ^Ptf1a^ mice (blue arrows). Bars: 1 mm.

To identify the gene causing *Sd*, we prepared two replacement vectors. One contained the *Ptf1a* ORF flanked by *loxKR3* and *lox*2272 [16], [18]. Using Cre-mediated recombination, we replaced the phosphoglycerate kinase 1 promoter (PGK)-*neo* gene with the *Ptf1a* ORF in ETn*-Gm13336-Ptf1a* ^neo^ ES cells (Figure 7A). The other replacement vector contained exons 1 and 2 of the *Gm13336* gene with a CAG promoter flanked by *loxKR3* and *lox*2272. Using Cre-mediated recombination, we replaced the PGK-*neo* gene with the CAG-exons 1 and 2 of *Gm13336* (CAG-*Gm13336*(1–2)) in ETn-*Gm13336-Ptf1a* ^neo^ ES cells (Figure 7A). Although we confirmed the expression of *Gm13336* and m*Gm13336* in ETn-*Gm13336-Ptf1a* ^Gm13336(1–2)^/+-+ and ETn-*Gm13336-Ptf1a* ^Gm13336(1–2)^/ETn-*Gm13336-Ptf1a* ^Gm13336(1–2)^ mice, we observed no abnormal phenotypes (Figure 7B). In contrast, the ETn-*Gm13336-Ptf1a* ^Ptf1a^/+-+ mice showed a short tail similar to that in *Sd/*+ mice, while ETn-*Gm13336-Ptf1a* ^Ptf1a^/ETn-*Gm13336-Ptf1a* ^Ptf1a^ neonates showed no tail and a short trunk (Figure 7B and Figure S6A). Histological examination revealed defects of the nucleus pulposus of the intervertebral discs and anorectal malformations similar to the defects in *Sd/Sd* mice, although the kidney defect was less severe (Figure 7C). As expected, the pancreas was restored to normal in ETn-*Gm13336*-*Ptf1a* ^Ptf1a^/ETn-*Gm13336-Ptf1a* ^Ptf1a^ mice (Figure 7C). The vertebral columns of ETn-*Gm13336-Ptf1a* ^Ptf1a^/+-+ and ETn-*Gm13336*-*Ptf1a* ^Ptf1a^/ETn-*Gm13336-Ptf1a* ^Ptf1a^ mice were usually truncated at the eighth caudal and the tenth thoracic vertebrae, respectively (Figure S6B).

To exclude the possibility that *Gm13336* or m*Gm13336* is responsible for *Sd*, we introduced CAG-*Gm13336* or CAG-m*Gm13336* into the gene-trap ES cell line 21-B137 (Figure S7A, Text S1). We confirmed the expression of these transcripts and observed no abnormalities (Figure S7B).

### Ectopic expression of *Ptf1a* from the ETn-*Gm13336*-*Ptf1a* allele

**Figure 8:**
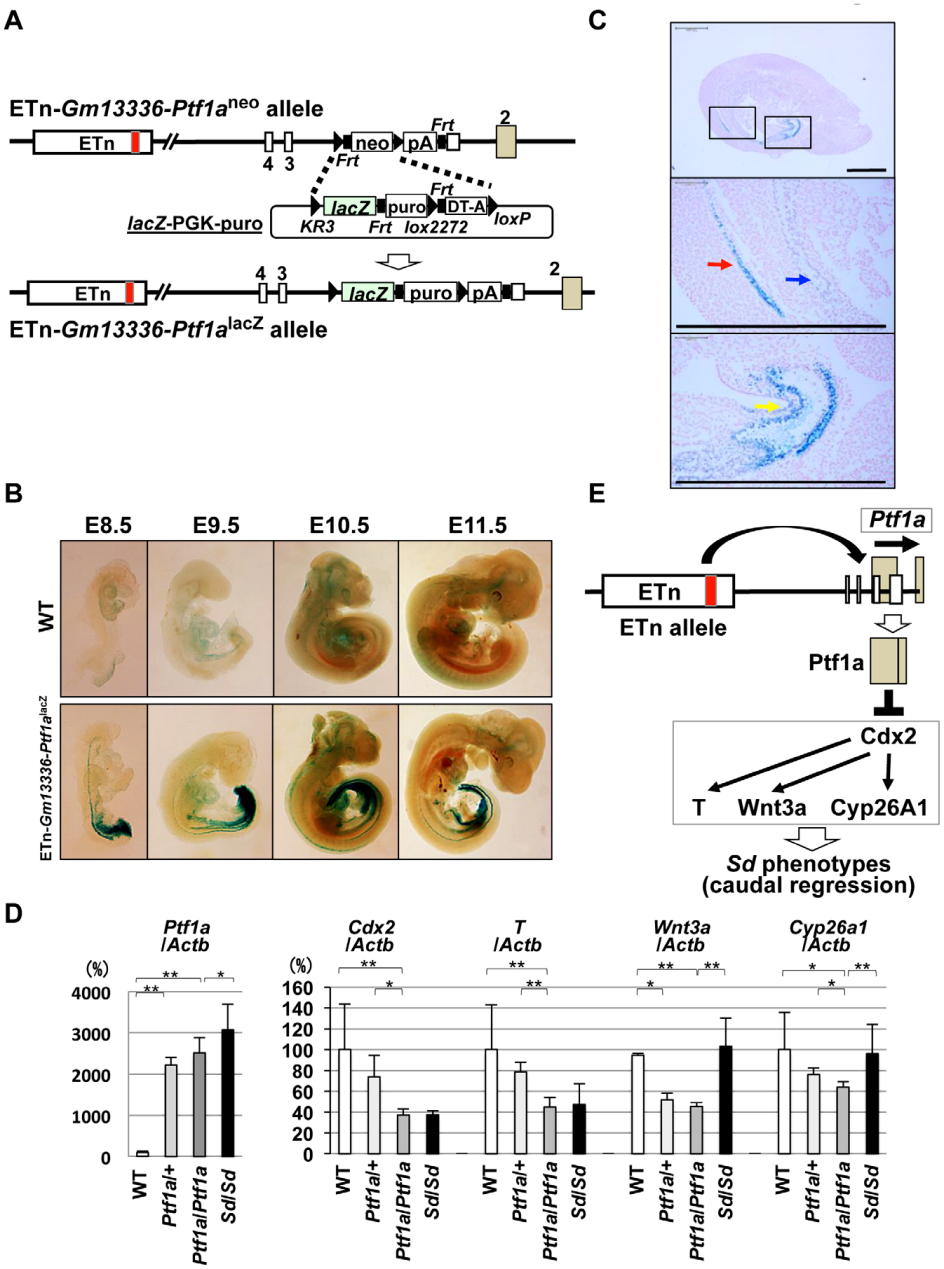
Ectopic expression of *Ptf1a* and downregulation of *Cdx2* and its downstream targets. A. Strategy for generating ETn-*Gm13336-Ptf1a* ^lacZ^ mice. The PGK-*neo* gene was replaced with the *lacZ* gene in the ETn-*Gm13336-Ptf1a* ^neo^ allele using Cre-mediated recombination. B. Whole-mount X-gal staining of ETn-*Gm13336-Ptf1a* ^lacZ^ embryos. Upper panel: wild-type (WT) littermates. Lower panel: ETn-*Gm13336Ptf1a* ^lacZ^ embryos. *LacZ* expression was detected in the notochord and hindgut at E8.5 to E9.5. Then, *lacZ* expression extended to the cloaca and mesonephros at E10.5 and to the pancreatic bud at E10.5 and E11.5. C. Ectopic *lacZ* expression. *LacZ* expression was strongly detected in the notochord (red arrow), mesonephros (blue arrow), and cloaca (yellow arrow) by histological analysis with X-gal staining at E9.5. The areas shown at higher magnification in the lower panels are indicated by boxes. Bars: 500 µm. D. Quantitative RT-PCR analyses in E9.5+/+, *Ptf1a*/+, and *Ptf1a*/*Ptf1a* littermates and in *Sd*/*Sd* embryos with a C57BL/6 genetic background. Although the expression of *Ptf1a* in ETn-*Gm13336-Ptf1a* ^Ptf1a^/*+-+* and homozygous ETn-*Gm13336-Ptf1a* ^Ptf1a^/ETn-*Gm13336-Ptf1a* ^Ptf1a^ mice was slightly lower than that in *Sd*/*Sd* mice, it was much higher than that in WT mice. The expression levels of *Cdx2 and T* in ETn-*Gm13336-Ptf1a* ^Ptf1a^/ETn-*Gm13336-Ptf1a* ^Ptf1a^ mice were similar to those in *Sd*/*Sd* mice, but significantly lower than those in WT mice. The expression of *Wnt3a* and *Cyp26a1* was decreased in ETn-*Gm13336-Ptf1a* ^Ptf1a^/ETn-*Gm13336-Ptf1a* ^Ptf1a^ mice, but not in *Sd*/*Sd* mice. The data represent the mean ± SD of independent whole embryos (+/+: *n* = 8, *Ptf1a*/+: *n* = 4, *Ptf1a*/*Ptf1a*: *n* = 6, *Sd*/*Sd*: *n* = 3–10). *p<0.05; ** *p*<0.01. E. Model of *Ptf1a* action. The ETn insertion induces ectopic expression of *Ptf1a*, resulting in downregulation of *Cdx2* and its downstream targets *T*, *Wnt3a*, and *Cyp26a1*. This combined deficiency of gene expression causes the *Sd* phenotype.

If the *Ptf1a* gene is responsible for *Sd*, *Ptf1a* should be expressed in tissues where the various phenotypes are observed. To examine the expression pattern of *Ptf1a* during embryonic development, we first tried to detect *Ptf1a* mRNA by in situ hybridization or PTF1a protein by immunohistochemistry. However, we failed to detect the expression of *Ptf1a* by either method, probably because of low-level expression and the low sensitivity of these methods. Thus, we used Cre-mediated recombination to replace the PGK-*neo* cassette in ETn-*Gm13336-Ptf1a* ^neo^ ES cells with the *lacZ* gene (Figure 8A). These mice were examined for *lacZ* expression by whole mount X-gal staining during embryonic development at E8.5, E9.5, E10.5, and E11.5 (Figure 8B). Interestingly, *lacZ* expression was detected in the notochord and hindgut at E8.5 and E9.5. *LacZ* expression extended to the cloaca and mesonephros at E10.5 and to the pancreatic bud at E10.5 and E11.5. *LacZ* expression was strongly detected in the notochord, mesonephros, and cloaca at E9.5 (Figure 8C).

Kawaguchi et al. previously reported that expression of *lacZ* in *Ptf1a* ^Cre^;ROSA26R mice was restricted to the pancreas during development in *Ptf1a*-Cre transgenic mice in which the *Cre* gene was inserted into the *Ptf1a* locus [19]. Therefore, this ectopic expression in ETn-*Gm13336-Ptf1a* ^lacZ^ embryos was considered to be induced by ETn insertion. Taken together, the ectopic expression pattern of *Ptf1a* is consistent with the phenotypes observed in *Sd*.

### Effect of ectopic *Ptf1a* expression

To examine the effects of ectopic *Ptf1a* expression, we carried out transcriptional profiling using E10.0 *Sd*/*Sd* and WT whole embryos (Tables S1 and S2) (Text S1). As expected, *Ptf1a* was upregulated 3.7-fold in *Sd*/*Sd* embryos. Among the significantly downregulated genes, we focused on the *Cdx2* and *T* genes, because *Cdx2* is known to regulate the expression of genes such as *T*, *Wnt3a*, and *Cyp26a1*, which are essential for development of the posterior embryo [20]. In fact, mice mutant for these genes show tail phenotypes similar to those in *Sd* mice [21], [22], [23], [24], [25], [26], [27]. Thus, the expression of these genes may be suppressed by the ectopic expression of *Ptf1a* in tissues such as the notochord, gut, and mesonephros. We analyzed the mRNA expression of *Ptf1a, Cdx2*, *T*, *Wnt3a*, and *Cyp26a1* in ETn-*Gm13336*-*Ptf1a* ^Ptf1a^/+-+ and ETn-*Gm13336*-*Ptf1a* ^Ptf1a^/ETn-*Gm13336*-*Ptf1a* ^Ptf1a^ embryos as well as in *Sd* E9.5 embryos. The level of *Ptf1a* expression in ETn-*Gm13336*-*Ptf1a* ^Ptf1a^/ETn-*Gm13336*-*Ptf1a* ^Ptf1a^ embryos was about 80% of that in *Sd* embryos, but was 20-fold higher than that in WT embryos (Figure 8D). As mentioned above, RNA profiling showed a 3.7-fold increase, but this discrepancy could be caused by differences in sensitivity and specificity. Both *Cdx2* and *T* expression in ETn-*Gm13336*-*Ptf1a* ^Ptf1a^/ETn-*Gm13336*-*Ptf1a* ^Ptf1a^ and *Sd* embryos decreased to about 40% of that in WT embryos at E9.5. *Wnt3a* and *Cyp26a1* expression was decreased to 42% and 62% of WT levels, respectively, in ETn-*Gm13336*-*Ptf1a* ^Ptf1a^/ETn-*Gm13336*-*Ptf1a* ^Ptf1a^ embryos, while their expression in *Sd*/*Sd* embryos was similar to that in WT embryos. However, only two genes, *Cdx2* and *T*, were downregulated at E10.0 and E11.5 in ETn-*Gm13336*-*Ptf1a* ^Ptf1a^/ETn-*Gm13336*-*Ptf1a* ^Ptf1a^ mice (Figure S8).

We further analyzed whether expression of *Ptf1a* can suppress the expression of *Cdx2*, *T*, *Wnt3a*, and *Cyp26a1* in ES cells. We inserted the CAG-*Ptf1a* gene into the 21-B137 allele using the same method (see Figure S9A). As expected, the mRNA expression of *Cdx2*, *T*, *Wnt3a*, and *Cyp26a1* was significantly decreased (Figure S9A). Furthermore, we transiently expressed *Ptf1a* in WT ES cells by electroporating in the expression vector CAG-*Ptf1a*. As shown in Figure S9B, the expression of *Cdx2*, *T*, *Wnt3a*, and *Cyp26a1* was decreased compared with that in ES cells transfected with the control CAG-enhanced green fluorescent protein (*EGFP*) (Text S1).

As suggested by our ES cell data shown in Figure S9, *Ptf1a* expression can downregulate the expression of four genes: *Cdx2*, *T*, *Wnt3a*, and *Cyp26a1*. Accordingly, all four genes were downregulated in E9.5 ETn-*Gm13336*-*Ptf1a* ^Ptf1a^/ETn-*Gm13336*-*Ptf1a* ^Ptf1a^ embryos (Figure 8D). However, only two genes, *Cdx2* and *T*, were downregulated at E10.0 and E11.5 (Figure S8), similar to the result observed at E9.5 in *Sd* embryos. The precise spatial or temporal regulation of *Ptf1a* gene expression in *Sd* mice could differ from that in ETn-*Gm13336*-*Ptf1a* ^Ptf1a^ mice because of the difference in allele structure.

Taken together, our results strongly suggest that ectopic expression of *Ptf1a* induced by ETn insertion suppresses the *Cdx2* gene and its downstream targets such as *T*, *Wnt3a*, and *Cyp26a1*, resulting in the characteristic phenotypes observed in *Sd* mice (Figure 8E).

## Discussion

In this study, we have revealed the nature of the *Sd* mutation to be the insertion of an ETn causing ectopic expression of *Ptf1a* in the caudal region of the embryo, resulting in suppression of *Cdx2* and its downstream target genes.

Endogenous retroviruses are present in the genomes of all vertebrates [28], [29]. Retrotransposons are genetic elements that can amplify themselves in a genome and are ubiquitous components of the DNA of many eukaryotic organisms. They are responsible for the majority of ERV-induced de novo germline mutations [30]. Most commonly, germline mutations caused by retrotransposon insertions occur in an intron, disrupting gene expression by causing premature polyadenylation, aberrant splicing, or ectopic transcription driven by the long terminal repeat. For ETn insertions, the most commonly reported defect is premature polyadenylation within the ETn, coupled with aberrant splicing because of a few commonly used cryptic splice signals [31]. However, ETn-promoted ectopic gene expression has not previously been observed [31]. Kano et al. reported that dactylaplasia (*Dac*) is a LTR retroptransposon insertion caused by the type D mouse endogenous provirus element (MusD), and that the ectopic MusD expression at the apical ectodermal ridge of limb buds correlates with the dactylaplasia phenotype [32]. However, in this *Dac* mutation, ETn-promoted ectopic expression of any endogenous genes has not been observed. In this study, we revealed the insertion of an ETn approximately 12 kb upstream of the transcription initiation site of the *Ptf1a* gene, resulting in the misexpression of *Ptf1a*. As the expression of *Gm13344* and *Gm13336* was also increased by this insertion, ETn acts as an enhancer, instead of as a transcription initiator. To the best of our knowledge, this is the first report of an ETn insertion causing ectopic gene expression.

*Ptf1a*, which encodes a basic helix-loop-helix transcription factor, was originally reported to be a pancreatic determiner that drives undifferentiated cells in the foregut endoderm to differentiate into a pancreatic lineage [19], [33]. In humans, *Ptf1a* was identified as responsible for the human permanent neonatal diabetes mellitus associated with cerebellar ataxia, and was reported to be involved in cerebellar development [34]. In fact, ectopic expression of *Ptf1a* conferred inhibitory gamma aminobutyric acid characteristics to neural progenitors, which were normally fated to become glutamatergic excitatory neurons [35]. These loss- and gain-of-function experiments suggested that Ptf1a itself acts as a cell fate determinant. However, in our case, *Ptf1a* overexpression and/or ectopic expression resulted in downregulation of *Cdx2* and its downstream targets *T*, *Wnt3a*, and *Cyp26a1*. The shortened tail phenotype of the *Sd* mutant is similar to that of the *Cdx2* mutant, although heterozygous *Cdx2* mutants show an anterior homeotic shift of the cervical and thoracic spine and rib abnormalities that are not observed in *Sd* mice [23]. The shortened tail phenotype of the *T* mutant [23], [36] is also similar to that of the *Sd* mutant. A mutation in the *Wnt3a* gene results in a lack of caudal somites, a disrupted notochord, and failure to form a tail bud [37], [38]. *Cyp26a1*-null mutants die during mid–late gestation and show a number of major morphogenetic defects, such as truncation of the tail, deficiencies of the external genitalia, anal atresia, and horseshoe kidneys [25], [26]. Interestingly, expression of *T* and *Wnt3a* in the tail bud was downregulated in *Cyp26a1*-deficient mice. Taken together, these results suggest that the phenotypes observed in *Sd* mice are caused by the combination of partial deficiency of *Cdx2* and its downstream target genes.

We demonstrated that the mRNA expression of *Cdx2*, *T*, *Wnt3a*, and *Cyp26a1* was significantly decreased in ES cells overexpressing *Ptf1a*. Ptf1 is a trimeric transcription factor comprising Ptf1a, an E protein (such as Tcf4 or Tcf12 [39]), and a third protein such as the mammalian Suppressor of Hairless (RBP-J) or its paralog RBP-L [40]. However, the binding site for Ptf1 has not been reported in the *Cdx2* gene, nor in the *T*, *Wnt3a*, or *Cyp26a1* genes. Instead, there are two Tcf-binding elements in the promoters of both the *Cdx2* and *T* genes in the mouse [41], [42]. It is possible that overexpressed Ptf1a can bind to Tcf4, preventing the binding of Tcf4 to the promoter, leading to attenuation of the expression of *Cdx2* and *T*. Decreased expression of *Cdx2* may cause activation of β-catenin-mediated transcriptional activity, because Cdx2 directly binds β-catenin and disrupts the β-catenin–Tcf protein complex [42], [43], [44]. However, nuclear β-catenin interacts with the Tcf/lymphoid enhancer factor (Lef) family of DNA-binding proteins to regulate the expression of numerous Wnt target genes [45], [46], [47]. Thus, the removal of Tcf by Ptf1a may result in the degradation of β-catenin and downregulation of the Wnt target genes.

CRS is a congenital heterogeneous constellation of caudal anomalies that includes varying degrees of agenesis of the spinal column, anorectal malformations, and genitourinary anomalies. Its pathogenesis is unclear, but it could be the result of excessive physiologic regression of the embryonic tail. As described above, the various mouse mutants have shown that caudal agenesis occurs a result of hypodevelopment of the anterior–posterior axis. The *Sd* mouse is considered a model for human CRS based on phenotypic similarity in the spine, hindgut, and urogenital system. The exact etiology in humans is unknown, except in some cases of Currarino syndrome. Currarino syndrome is a form of CRS with hemisacrum, anorectal malformations, and presacral mass, such as teratoma [48]. Previous reports have shown that *HLXB9* is a major causative gene for Currarino syndrome [49], [50]. HLXB9 is a homeobox protein that contains a glycine-and-alanine-rich region and a strongly acidic region next to the amino-terminal and carboxy-terminal side of the homeodomain, respectively [51]. However, a null mutation of the *Hlxb9* gene in mice showed agenesis of the dorsal pancreas but no skeletal truncation [52], [53]. It is of interest that both *Ptf1a* and *Hlxb9* are expressed in the pancreas, and that *Ptf1a* is required for *Hlxb9* expression [54]. Although it is not clear whether *PTF1A* is involved in human CRS, the evidence from this study implicates *PTF1A* and possibly *HLXB9* in the caudal abnormalities. Further studies using *Sd* mice will provide further insight into the development of human CRS.

## Materials and Methods

### *Danforth's short tail* (*Sd*) mice

*Sd* mice purchased from the Jackson Laboratory (Bar Harbor, ME) were backcrossed to C57BL/6 mice for at least ten generations. All experiments were performed in accordance with the Declaration of Helsinki and were approved by the Kumamoto University Ethics Committee for Animal Experiments (authorization number in Kumamoto University: C23-262, C24-278).

### Identification of *Sd* mice by PCR and Southern blotting

PCR was used to genotype *Sd* alleles. For the WT allele, the 5′ primer 5′Sd-S1 (5′-GAAAGCAAAGGGCTGCTTAC-3′) and the 3′ primer 3′Sd-A1 (5′-TATTCTTGCAGGGAGAGTTG-3′) were used to amplify a 283-bp fragment. To detect the *Sd* allele, the 5′ primer 5′Sd-S1 (5′-GAAAGCAAAGGGCTGCTTAC-3′) and the 3′ primer 5′Tn-A1 (5′-TCTCGTGTGATCTGTCTGTC-3′), located in the ETn, were used to amplify a 228-bp fragment. The PCR conditions were as follows: denaturation at 94°C; followed by 30 cycles of denaturation at 94°C for 30 s, annealing at 56°C for 30 s, and extension at 72°C for 30 s. PCR products were visualized on a 1% agarose gel using ethidium bromide. For Southern blotting, genomic DNA was digested overnight with Sph I and subjected to electrophoresis on a 1% agarose gel. DNA was transferred onto a positively charged nylon membrane (Roche, Indianapolis, IN). After baking at 80°C for 1 h, the membrane was hybridized with a flanking genomic DNA-specific probe (Figure 2A).

### ES cell culture

ES cells were cultured in KSR-GMEM medium consisting of Glasgow Minimum Essential Medium (Sigma, St Louis, MO) with 1×nonessential amino acids (Gibco Invitrogen, Grand Island, NY), 0.1 mM β-mercaptoethanol, 1 mM sodium pyruvate, 1% fetal bovine serum (HyClone; Thermo Fisher Scientific Inc., Waltham, MA), 14% Knockout Serum Replacement (Gibco Invitrogen), and 1100 U/ml leukemia inhibitory factor (ESGRO; Chemicon, Temecula, CA).

### Generation of ETn knock-in mice

The knock-in method used in this study was developed by us and has been described [16], [17], [18]. In the first step, the targeting vector—containing a 5′ homology region, *loxP*, *Frt*, PGK-*neo* cassette, *lox2272*, polyadenylation signal (pA), *Frt*, 3′ homology region, and an MC1 promoter-diphtheria toxin A fragment with a pA (MC1-DT-A)—was constructed using pBluescript II containing the PGK-*neo* cassette (p03) [55]. Targeting vectors were electroporated into feeder-free KTPU8 ES cell lines derived from the TT2 ES cell line, according to previously described methods [56], [57]. Three targeted ES clones were obtained from 288 G418-resistant clones. ES cells were aggregated with ICR morulas to produce chimeric mice. Germline transmission was confirmed in all three lines. In the second step, the replacement vector—containing *loxKR3* (*KR3*), ETn, *Frt*, puromycin *N*-acetyl-transferase (*puro*), *lox2272*, *Frt*, MC1-DT-A, and *loxP*—was electroporated into the targeted ES cell clones to establish the kiETn allele [18].

The PGK-*puro* cassette was removed by transient Flp expression. One replaced ES clone was obtained from 28 puromycin-resistant clones. These kiETn ES cells were used to produce germline chimeras. Heterozygous kiETn mice were backcrossed to C57BL/6 for at least five generations. Then, heterozygous kiETn mice were intercrossed to produce homozygous kiETn mice.

### Identification of kiETn mice

Founder kiETn mice were identified by PCR and Southern blotting. Genomic DNA was extracted from an ear clip. The ETn fragment digested by Xba I contained both 5′ and 3′ flanking genomic sequences; partial tandem duplication (5′: 287-bp; 3′: 630-bp) of flanking genomic sequences was confirmed by PCR analyses. To detect the kiETn allele, the 5′ primer 5′Sd-S1 (5′-GAAAGCAAAGGGCTGCTTAC-3′), located in the 5′ flanking genomic region, and the 3′ primer 5′Tn-A1 (5′-TCTCGTGTGATCTGTCTGTC-3′), located in the ETn, generated 228-bp and 650-bp fragments, respectively. To detect the WT allele, the 5′ primer 5′Sd-S1 (5′-GAAAGCAAAGGGCTGCTTAC-3′), located in the 5′ flanking genomic region, and the 3′ primer 3′Sd-A1 (5′-TATTCTTGCAGGGAGAGTTG-3′), located in the 3′ flanking genomic region, generated a 283-bp fragment. To detect the targeted allele, the 5′ and 3′ primers neo-F (5′-AGAGGCTATTCGGCTATGAC-3′) and neo-R (5′-CACCATGATATTCGGCAAGC-3′), respectively, both located in the targeting vector, generated a 545-bp fragment. For Southern blotting, genomic DNA was digested overnight with Sph I and subjected to electrophoresis on a 1.0% agarose gel. DNA was transferred onto a positively charged nylon membrane (Roche). After baking at 80°C for 1 h, the membrane was prehybridized and then hybridized using flanking genomic DNA-specific probes (Figure 2A) prepared using a Digoxigenin DNA Labeling and Detection Kit (Roche).

### RT–PCR

PCR amplification of each DNA fragment and gene was performed using TaKaRa EX or LA Taq (Takara, Kyoto, Japan), according to the manufacturer's protocol. The primer sequences were as follows: *Gm13344* (accession number AB701678 and AB701679): 5′-ACGAATGGGGTGTTCAGACG-3′ (sense) and 5′-CGACTGCCAGACCCAGGAAG-3′ (antisense), generating two alternative splicing products, 446-bp and 297-bp fragments; *Gm13336* (accession number AB701680): 5′-TGACGCTTTGTGAGTGATCC-3′ (sense) and 5′-AACACTCCTGTGATGTGTAG-3′ (antisense), generating a 226-bp fragment; m*Gm13336*: 5′-TGACGCTTTGTGAGTGATCC-3′ (sense) and 5′-GAACAATACGATTTCTTTTTACCTG-3′ (antisense), generating a 488-bp fragment; *Ptf1a*: 5′-TGAGGGACCTACCCGAATTG-3′ (sense) and 5′-ACAATATGCACAAAGACGCG-3′ (antisense), generating a 1,105-bp fragment; *Actb* (*β-actin*; control): 5′-ATGTACGTAGCCATCCAGGC-3′ (sense) and 5′-AAGAAGGAAGGCTGGAAAAG-3′ (antisense), generating a 407-bp fragment; *Gapdh* (glyceraldehyde 3-phosphate dehydrogenase; control): 5′-GGAAAGCTGTGGCGTGATG-3′ (sense) and 5′-CTGTTGCTGTAGCCGTATTC-3′ (antisense), generating a 392-bp fragment. Thermal cycling was carried out with denaturation at 94°C; followed by 30, 35, or 40 cycles of denaturation at 94°C for 30 s, annealing at 56°C for 30 s, and extension at 72°C for 30 s. PCR products were visualized on 1% agarose gels using ethidium bromide.

### Quantitative real-time RT–PCR

Reverse transcribed products were used for quantitative real-time PCR using an Applied Biosystems 7500 Real-Time PCR system (Applied Biosystems, Foster City, CA). TaqMan Gene Expression Master Mix and TaqMan Gene Expression Assays for *Gm13336* (a custom-made *Gm13336* TaqMan probe), m*Gm13336* (a custom-made m*Gm13336* TaqMan probe), *Ptf1a* (Mm 00479622), *Cdx2* (Mm 01212280), *T* (Mm 01318252), *Wnt3a* (Mm 00437337), *Cyp26a1* (Mm 00514486), and *Actb* (Mm 00607939) were purchased from Applied Biosystems. Reactions were carried out under the following conditions: 2 min at 50°C and 10 min at 95°C; followed by 40 cycles of 15 s at 95°C and 1 min at 60°C. Calibration was conducted using the relative standard curve method. To construct a standard curve, a standard sample cDNA was prepared from E10.5 embryos for *Cdx2*, *T*, *Wnt3a*, *Cyp26a1*, and *Actb,* or *Ptf1a* cDNA for *Ptf1a,* or *Gm13336* cDNA for *Gm13336*, or m*Gm13336* cDNA for m*Gm13336*. For each PCR assay, the standard curve was generated using the same standard sample. The relative concentration of the target gene in each sample was calculated from the constructed standard curve, and the ratio of the relative concentration of the target gene to *Actb* in each sample was calculated. This ratio represented the relative expression of the target gene normalized to *Actb* compared with the standard sample. *Actb* is recommended by Applied Biosystems as a suitable endogenous internal control for TaqMan RT-PCR analyses. Real-time PCR for *Gm13344* transcripts was performed using the THUNDERBIRD SYBR qPCR mix (Toyobo, Osaka, Japan). The following primer pair was used for the shorter splice variant *Gm13344* transcripts: 5′-TGTGCTGGACCCAAACATAGCCAAAG-3′ (sense) and 5′-CGACTGCCAGACCCAGGAAG-3′ (antisense), generating a 293-bp fragment. The relative concentration of *Gm13344* in each sample was calculated from the constructed standard curve, and the ratio of the relative concentration of *Gm13344* to *Actb* was calculated. This ratio represented the relative expression of the target gene normalized to *Actb* compared with the standard sample.

### Generation of transgenic mice

To isolate the 21,815-bp DNA fragment containing *Gm13344*-ETn and the 24,714-bp DNA fragment containing ETn-*Gm13336* from a cosmid clone, cosmid DNA was digested with Xho I and Bsm BI, respectively. Isolated DNA fragments were microinjected into fertilized eggs obtained from C57BL/6 mice, at a final concentration of 1 µg/ml in Tris-EDTA buffer.

### Targeting vector construction and generation of ETn-*Gm13336-Ptf1a* ^neo^ mice

Genomic DNA containing the coding region of the *Ptf1a* gene was isolated from the cosmid clone containing the ETn. The pBluescript II construct containing the PGK-*neo* cassette (p03) and the MC1-DT-A fragment was used as a backbone to construct the targeting vector. The targeting vector comprised a 5′ homology region, *loxP*, *Frt*, PGK-*neo*, *lox2272*, pA, *Frt*, 3′ homology region, and MC1-DT-A (Figure 6A). The 5′ homology region contained the first exon, first intron, second exon, and part of the second intron of *Gm13336*; part of the first exon of *Ptf1a* was deleted by insertion of the *neo* cassette. The targeting vector was electroporated into *Sd*/+ ES cells as described above. Nine targeted ES clones were obtained from 192 G418-resistant clones. ES cells were aggregated with ICR morulas as described above. Germline chimeras were obtained from four ES lines. This strain of mouse was designated ETn-*Gm13336-Ptf1a* ^neo^. ETn-*Gm13336-Ptf1a* ^neo^/+-+ mice were backcrossed to C57BL/6 mice for at least three generations. Then, ETn-*Gm13336-Ptf1a* ^neo^/+-+ mice were intercrossed to produce ETn-*Gm13336-Ptf1a* ^neo^/ETn-*Gm13336-Ptf1a* ^neo^ mice.

### Identification of ETn-*Gm13336-Ptf1a* ^neo^ mice

Founder ETn-*Gm13336-Ptf1a* ^neo^ mice were identified by PCR and Southern blotting. Genomic DNA was extracted from an ear clip. To detect the targeted allele, the 5′ primer neo-F (5′-AGAGGCTATTCGGCTATGAC-3′) and the 3′ primer neo-R (5′-CACCATGATATTCGGCAAGC-3′), located in the *neo* cassette, generated a 545-bp fragment. To detect the WT allele, the 5′ primer 5′AKKO-S1 (5′-ATTGCTCAGAACCCCTAGGG-3′), located in the 5′ flanking genomic region of the second exon of *Ptf1a*, and the 3′ primer 3′AKKO-A1 (5′-GATTCCCTGAGCTGTGAAGC-3′), located in the 3′ flanking genomic region of the second exon of *Gm13336*, generated a 1,777-bp fragment. For Southern blotting, genomic DNA was digested overnight with Eco RI and Spe I and electrophoresed on 1.0% agarose gels. DNA was transferred onto a positively charged nylon membrane (Roche). After baking at 80°C for 1 h, the membrane was prehybridized and then hybridized using 5′ and 3′ flanking genomic DNA-specific probes (Figure 6A) prepared using a Digoxigenin DNA Labeling and Detection Kit (Roche).

### Construction of the *Ptf1a* ORF replacement vector and establishment of ETn-*Gm13336-Ptf1a* ^Ptf1a^ ES cells and mouse lines

To insert the *Ptf1a* ORF into the ETn-*Gm13336-Ptf1a* allele, ETn-*Gm13336-Ptf1a^neo^* ES cells were used. The ES cell clones were electroporated with the Cre expression vector and a replacement vector assembled from p*KR3*-*Frt*-del.pA-*puro*-2272 with the cloned *Ptf1a* ORF to establish ETn-*Gm13336-Ptf1a* ^Ptf1a^ ES cell and mouse lines (Figure 7A). ETn-*Gm13336*-*Ptf1a* ^Ptf1a^ mice were backcrossed to C57BL/6 mice for at least three generations. *Gm13336*-*Ptf1a* ^Ptf1a^/+-+ mice were intercrossed to produce *Gm13336-Ptf1a* ^Ptf1a^/*Gm13336*-*Ptf1a* ^Ptf1a^ mice.

### Construction of CAG-*Gm13336*(1–2) and establishment of ETn-*Gm13336*-*Ptf1a* ^CAG-Gm13336(1–2)^ ES cells and mouse lines

To insert the CAG-*Gm13336*(1–2) into the ETn-*Gm13336-Ptf1a^neo^* allele, ETn-*Gm13336-Ptf1a^neo^* ES cells were used. The ES cell clones were electroporated with the Cre expression vector and a replacement vector assembled from p*KR3*-*Frt*-del.pA-*puro*-*2272* with the cloned *Gm13336*(1–2) to establish ETn-*Gm13336-Ptf1a* ^CAG-Gm13336(1–2)^ ES cell and mouse lines (Figure 7A). ETn-*Gm13336*-*Ptf1a* ^CAG-Gm13336(1–2)^ mice were backcrossed to C57BL/6 mice for at least three generations. Then, *Gm13336*-*Ptf1a* ^CAG-Gm13336(1–2)^/+-+ mice were intercrossed to produce *Gm13336-Ptf1a* ^CAG-Gm13336(1–2)^/*Gm13336*-*Ptf1a* ^CAG-Gm13336(1–2)^ mice.

### Construction of the *lacZ* replacement vector and establishment of the ETn-*Gm13336*-*Ptf1a^lacZ^* mouse line

ETn-*Gm13336*-*Ptf1a* ^neo^ ES cells were used to insert *lacZ* into the ETn-*Gm13336*-*Ptf1a* allele. The ES cell clones were electroporated with a replacement vector assembled from p*KR3*-*Frt*-del.pA-*puro*-*2272* and cloned *lacZ* to establish ETn-*Gm13336-Ptf1a* ^lacZ^ mouse lines (Figure 8A). ETn-*Gm13336-Ptf1a* ^lacZ^ mice were backcrossed to C57BL/6 mice for at least two generations.

### Histological analysis

Embryos and neonates were fixed in phosphate-buffered 15% formaldehyde overnight, rinsed twice for 1 h in phosphate-buffered saline (PBS), dehydrated through increasing concentrations of ethanol, equilibrated with xylene, embedded in paraffin wax, and sectioned at 4 µm. Sagittal sections were stained with hematoxylin and eosin and examined by light microscopy.

### Detection of β-galactosidase (*lacZ)* activity

Samples were fixed for 30 min at room temperature in fix solution [1% formaldehyde, 0.2% glutaraldehyde, and 0.02% NP-40 in PBS]. Fixed samples were washed twice with PBS and incubated overnight at 30°C in staining solution (5 mm potassium ferricyanide, 5 mm potassium ferrocyanide, 2 mm MgCl_2_, 0.5% X-gal in PBS). Samples were rinsed twice in PBS and then post-fixed in 10% formaldehyde. For observation of whole-mount X-gal staining, samples were made transparent using benzylalcohol/benzylbenzoate (1∶2), after dehydration with a series of ethanol steps (25%, 50%, 70%, 100%, and 100%, 1 h each). For histological analysis, samples were sectioned at 8 µm and counterstained with Nuclear Fast red (Funakoshi, Tokyo, Japan) after X-gal staining.

### Statistical analysis

The results are presented as the mean ± standard deviation (SD) of independent experiments as detailed separately in each corresponding figure legend. Data were compared using the Student's *t*-test and were considered significantly different at *p*<0.05.

## Supporting Information

Figure S1 Cosmid clones and PCR products covering the *Sd* locus. The top panel shows a genetic map of the *Sd* region, including the position of the proximal marker *D2Mit362* and the distal marker *Skt^Gt^*. The *Sd* region contains a minimum of seven genes; we assigned these genes to individual cosmid clones (C, open boxes) or PCR products (P, black boxes). The red arrowheads indicate the insertion point of the early transposon endogenous retrovirus 3 (ETn).
(PDF)

Figure S2 Details of cosmid clones and PCR products. A. Size of cosmid inserts and PCR products. The cosmid C3, shown in red, contains the ETn. B. DNA electrophoresis (Not I digestion) to measure the size of the cosmid clone inserts. The insert of cosmid clone C3 (shown in red) was bigger than the expected size based on its end-sequence tags and wild-type genome informatics. C. DNA electrophoresis (Not I-digestion) of the C3 cosmid clone only. This clone gave bands of 21,894-bp, 10,093-bp, 3,037-bp, and 1,416-bp (the precise band sizes were obtained after subsequent shotgun sequencing). Its total insert size (36,440-bp) was bigger than the expected size (27,936-bp). This was because of the difference in size of the largest band: 21,894-bp in the *Sd*-derived cosmid C3 and 13,390-bp in the wild type C57BL/6.
(PDF)

Figure S3 Establishment of neomycin-resistant (*neo*) mice. A. Wild-type (WT) and *neo* alleles. Cleavage at Sph I sites was used to distinguish between the two alleles. The blue and red bars indicate a fragment detected by Southern blotting for the WT allele and *neo* allele, respectively. The probe is shown as a black box. Primer pairs (5′Sd-S1/3′Sd-A1 and 5′Sd-S1/neo-A1) for PCR-based genotyping of the WT allele and *neo* allele, respectively, are shown as closed blue arrows and red arrows. The red arrowhead indicates the insertion point of the *neo* cassette. B. Genotyping by PCR (left) and Southern blotting (right). In the PCR, *neo*/+ mice carry both products, while WT (+/+) and *neo*/*neo* mice carry one of the two. By Southern blotting, *neo*/+ mice display two bands, while WT (+/+) and *neo*/*neo* mice show one of the two. C. Hematoxylin and eosin staining of the thoracic intervertebral discs and kidneys from *neo*/*neo* adult mice. These mice survive to adulthood and show no abnormalities in these tissues. Bars: 1 mm.
(PDF)

Figure S4 Establishment of *Sd*/+ ES cell clones. A. Genotyping of ES cell lines. ES cell lines were established from blastocysts obtained from a mating between an *Sd*/+ heterozygote and a wild-type mouse. In this figure, four lines were positive for the ETn allele and three of the four were positive for *Sry*, meaning that three were male *Sd*/+ ES cell lines. B. Short tail in chimeric mouse.
(PDF)

Figure S5 Establishment of ETn-*Gm13336/Ptf1a^neo^* mice. A. Upper panel: PCR-based detection of the ETn. Lower panel: PCR-based detection of the *neo* allele. Both the ETn and *neo* were transmitted to the offspring, suggesting that the ETn and *Gm13336-Ptf1a* ^neo^ are on the same chromosome. B. Hematoxylin and eosin staining of pancreases in E18.5 embryos showed no pancreas development in ETn*-Gm13336-Ptf1a* ^neo^/ETn*-Gm13336-Ptf1a* ^neo^ mice. Bars: 200 µm.
(PDF)

Figure S6 Morphology of tail of ETn-*Gm13336/Ptf1a* ^Ptf1a^ neonates. A. The ETn-*Gm13336-Ptf1a* ^Ptf1a^/+-+ neonates showed a short tail similar to that of *Sd* heterozygotes, while the ETn-*Gm13336-Ptf1a* ^Ptf1a^/ETn-*Gm13336-Ptf1a* ^Ptf1a^ neonates showed no tail and a short trunk. B. Histological examination revealed that the vertebral columns of ETn-*Gm13336-Ptf1a* ^Ptf1a^/+-+ and ETn-*Gm13336-Ptf1a* ^Ptf1a^/ETn-*Gm13336-Ptf1a* ^Ptf1a^ neonates were truncated at the eighth caudal (black arrows) and the tenth thoracic (red arrows) vertebrae, respectively. Black arrows and red arrows indicate the level of the terminal vertebral body for heterozygotes and homozygotes, respectively. Bars: 2 mm.
(PDF)

Figure S7 Generation and tail morphology of *Gm13336*-mutant mice. A. Strategy for insertion of the *Gm13336* and mutant (m) *Gm13336* gene into the 21-B137 locus. Normal *Gm13336* cDNA and m*Gm13336* cDNA driven by a CAG promoter was inserted into the 21-B137 locus using Cre-mediated recombination. B. Morphology of the tail in adult CAG-*Gm13336* and CAG-m*Gm13336* mice. The tail phenotype was normal.
(PDF)

Figure S8 Ectopic expression of *Ptf1a* and downregulation of *Cdx2* and its downstream targets. A. Quantitative RT-PCR analyses of the expression of *Ptf1a*, *Cdx2*, *T*, *Wnt3a*, and *Cyp26a1* in the E10.0 embryos of WT, *Ptf1a*/+, and *Ptf1a*/*Ptf1a* littermates. Upregulation of *Ptf1a* and downregulation of *Cdx2* and *T,* but not of *Wnt3a* and *Cyp26a1* were observed. The data represent the mean ± SD of independent whole embryos (+/+: *n* = 4, *Ptf1a*/+: *n* = 6, *Ptf1a*/*Ptf1a*: *n* = 3). *p<0.05; **p<0.01. B. Quantitative RT-PCR analyses of the expression of *Ptf1a*, *Cdx2*, *T*, *Wnt3a*, and *Cyp26a1* in E11.5 ETn-*Gm13336*/*Ptf1a* ^Ptf1a^ embryos of WT, *Ptf1a*/+, and *Ptf1a*/*Ptf1a* littermates. Upregulation of *Ptf1a* and downregulation of *Cdx2* and *T* were observed. The data represent the mean ± SD of three independent whole embryos. *p<0.05; **p<0.01.
(PDF)

Figure S9 Overexpression of *Ptf1a* attenuates the expression of *Cdx2* and its downstream targets. A. Quantitative RT-PCR analyses in ES cells with stable expression of *Ptf1a*. Expression of *Cdx2*, *T*, *Wnt3a*, and *Cyp26a1* was suppressed by stable overexpression of *Ptf1a*. The data represent the mean ± SD of independent cultures (B137: *n* = 4, CAG-*Ptf1a*: *n* = 6). **p<0.01. B. Quantitative RT-PCR analyses in ES cells transfected with a CAG-*EGFP* expression vector (white bars) or a CAG-*Ptf1a* expression vector (black bars). Expression of *Cdx2*, *T*, *Wnt3a*, and *Cyp26a1* was suppressed by transient overexpression of *Ptf1a*. The data represent the means ± SD of six independent cultures. *p<0.05; **p<0.01.
(PDF)

Table S1 Genes upregulated more than 1.7-fold in homozygous *Sd* embryos at embryonic day 10.0.
(PDF)

Table S2 Genes downregulated more than 1.7-fold in homozygous *Sd* embryos at embryonic day 10.0.
(PDF)

Text S1 Supplementary Materials and Methods. Cosmid library of *Sd* homozygotes, DNA sequencing, Extraction and reverse transcription of RNA, Cloning of the *Gm13336* cDNA, Skeletal preparations, X-ray computed tomography, Establishment of an *Sd*/+ ES cell line, Construction of replacement vectors for CAG-*Gm13336* and CAG-m*Gm13336*, and establishment of Ayu21-B137^CAG-Gm13336^ and Ayu21-B137^CAG-mGm13336^ mouse lines, Transfection of CAG-*Ptf1a* or CAG-*EGFP* expression vectors into ES cells, Microarray analysis methods are provided.
(DOCX)
