# Supplementary figures and images for: Ectopic Expression of Ptf1a Induces Spinal Defects, Urogenital Defects, and Anorectal Malformations in Danforth's Short Tail Mice
Source: PLoS Genet. 2013 Feb 21;9(2):e1003204. doi: 10.1371/journal.pgen.1003204 (PMC3578775; doi:10.1371/journal.pgen.1003204)

Figure S1

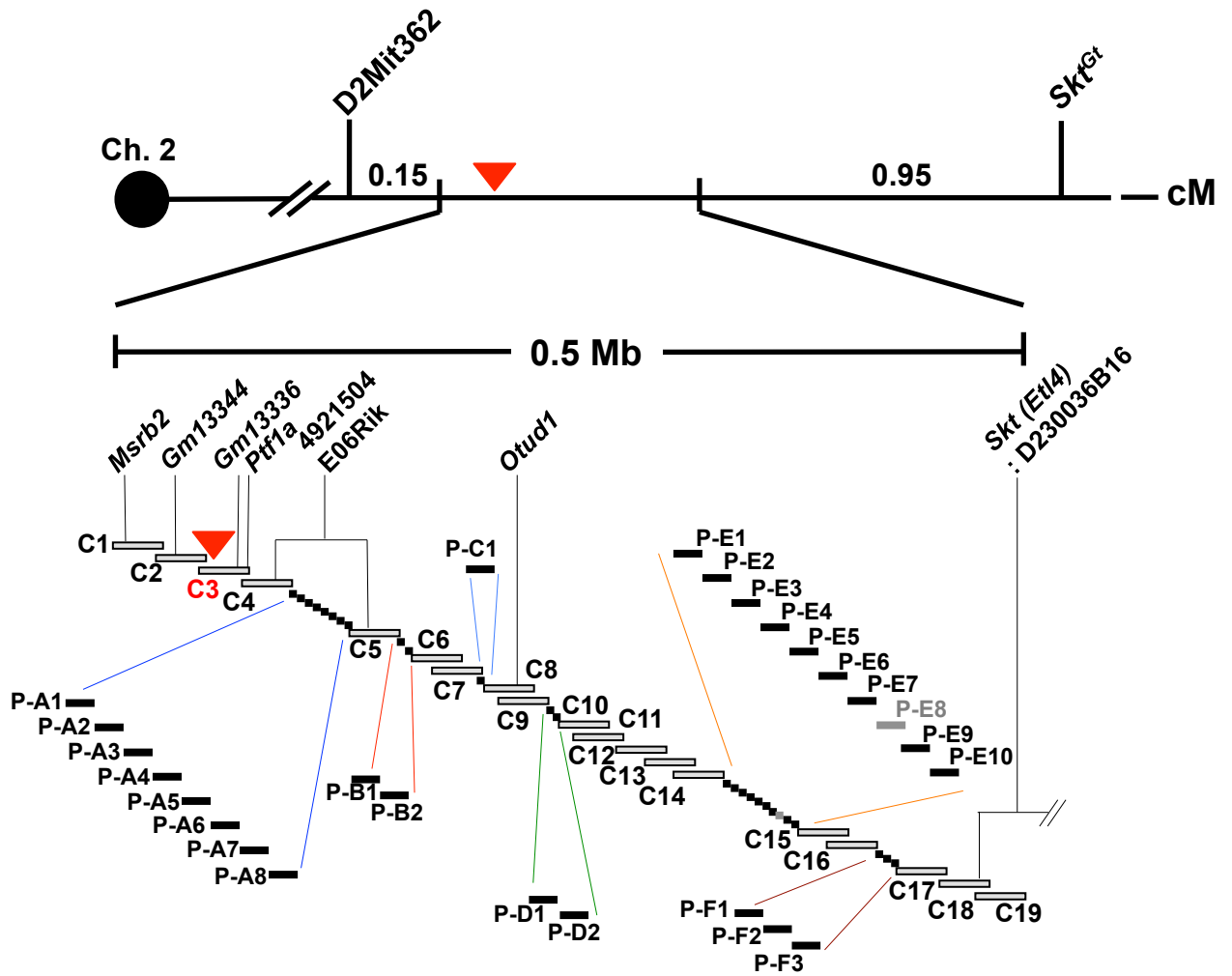

Supplement: Figure S1 — Cosmid clones and PCR products covering the Sd locus. The top panel shows a genetic map of the Sd region, including the position of the proximal marker D2Mit362 and the distal marker SktGt. The Sd region contains a minimum of seven genes; we assigned these genes to individual cosmid clones (C, open boxes) or PCR products (P, black boxes). The red arrowheads indicate the insertion point of the early transposon endogenous retrovirus 3 (ETn). (PDF) [file pgen.1003204.s001.pdf]

**Figure S3**

**A**

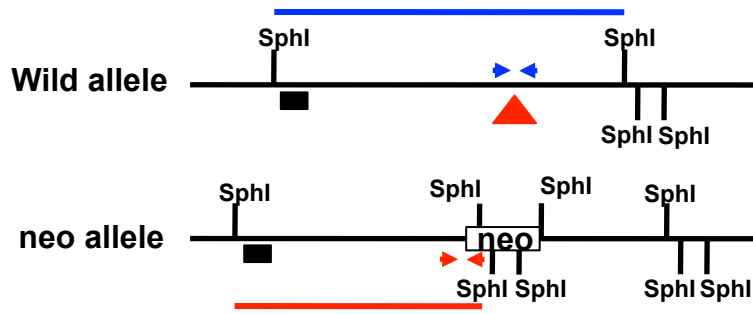

**B**

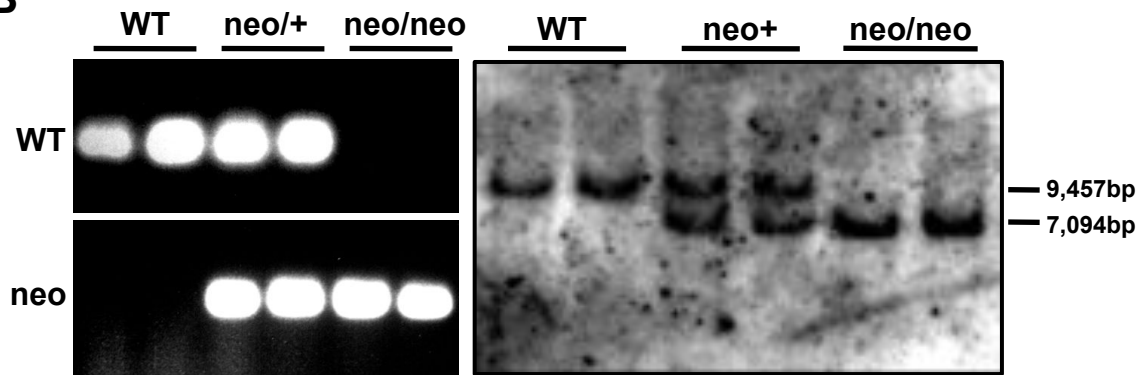

**C**

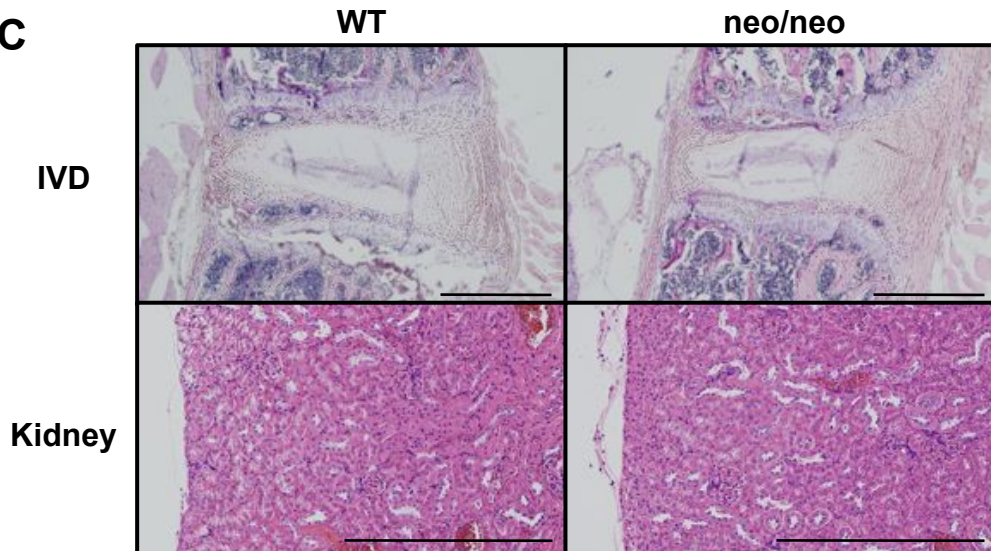

Supplement: Figure S3 — Establishment of neomycin-resistant (neo) mice. A. Wild-type (WT) and neo alleles. Cleavage at Sph I sites was used to distinguish between the two alleles. The blue and red bars indicate a fragment detected by Southern blotting for the WT allele and neo allele, respectively. The probe is shown as a black box. Primer pairs (5′Sd-S1/3′Sd-A1 and 5′Sd-S1/neo-A1) for PCR-based genotyping of the WT allele and neo allele, respectively, are shown as closed blue arrows and red arrows. The red arrowhead indicates the insertion point of the neo cassette. B. Genotyping by PCR (left) and Southern blotting (right). In the PCR, neo/+ mice carry both products, while WT (+/+) and neo/neo mice carry one of the two. By Southern blotting, neo/+ mice display two bands, while WT (+/+) and neo/neo mice show one of the two. C. Hematoxylin and eosin staining of the thoracic intervertebral discs and kidneys from neo/neo adult mice. These mice survive to adulthood and show no abnormalities in these tissues. Bars: 1 mm. (PDF) [file pgen.1003204.s003.pdf]

**Figure S4**

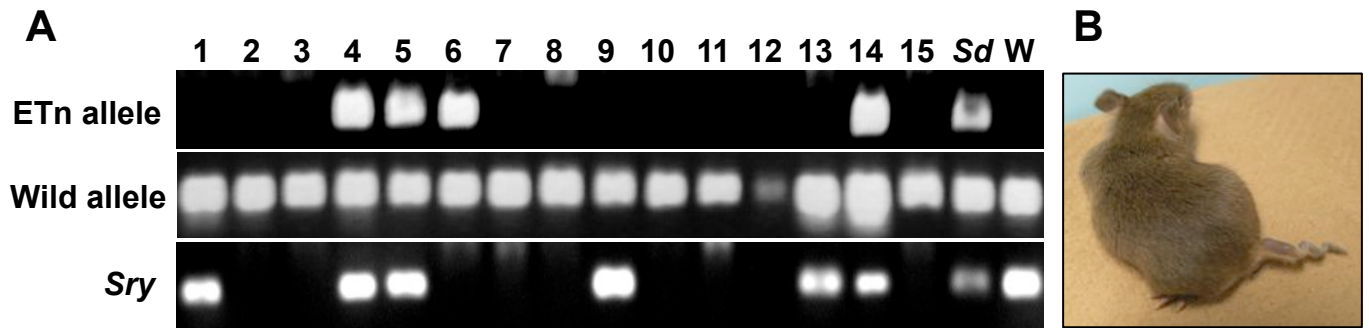

Supplement: Figure S4 — Establishment of Sd/+ ES cell clones. A. Genotyping of ES cell lines. ES cell lines were established from blastocysts obtained from a mating between an Sd/+ heterozygote and a wild-type mouse. In this figure, four lines were positive for the ETn allele and three of the four were positive for Sry, meaning that three were male Sd/+ ES cell lines. B. Short tail in chimeric mouse. (PDF) [file pgen.1003204.s004.pdf]

Figure S5

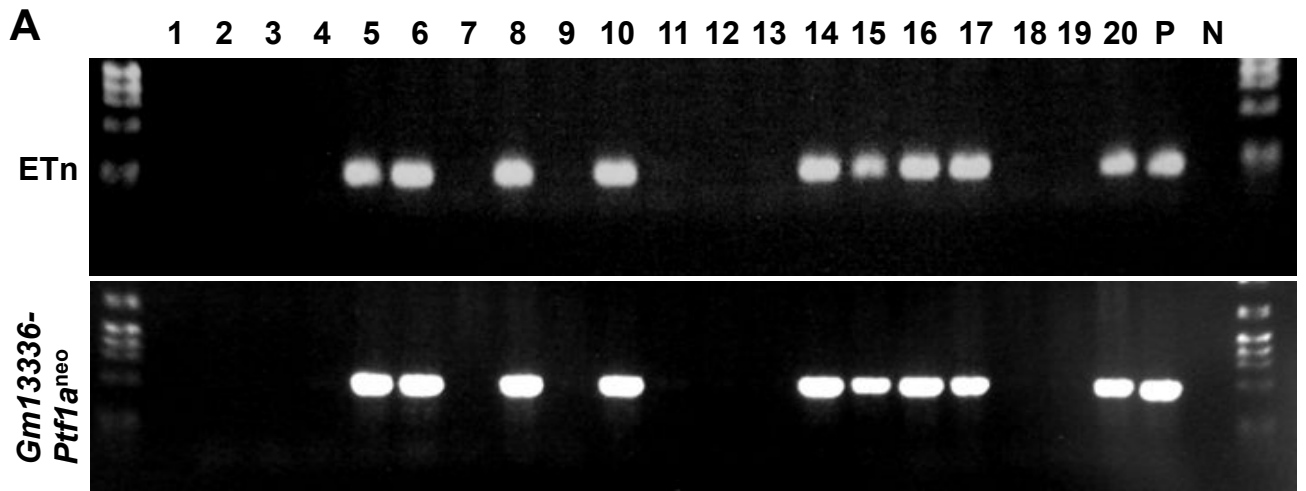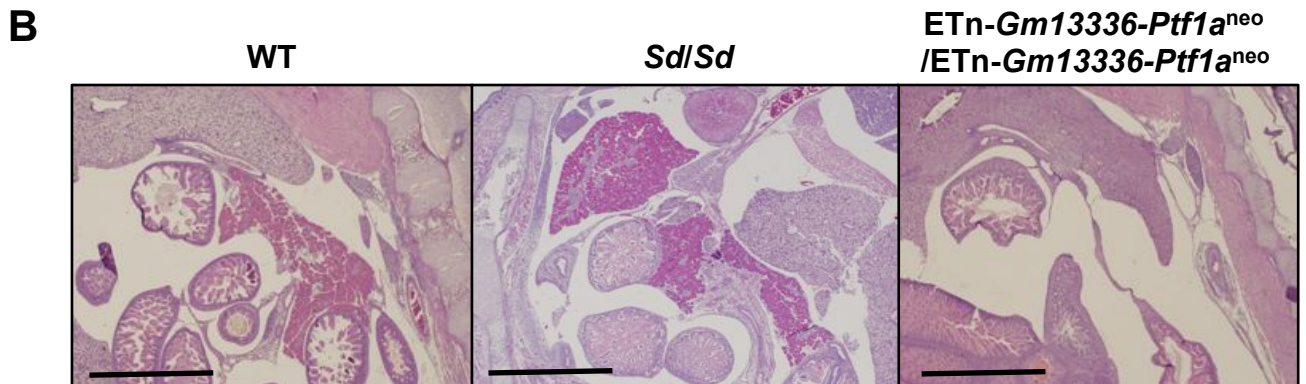

Supplement: Figure S5 — Establishment of ETn-Gm13336/Ptf1aneo mice. A. Upper panel: PCR-based detection of the ETn. Lower panel: PCR-based detection of the neo allele. Both the ETn and neo were transmitted to the offspring, suggesting that the ETn and Gm13336-Ptf1a neo are on the same chromosome. B. Hematoxylin and eosin staining of pancreases in E18.5 embryos showed no pancreas development in ETn-Gm13336-Ptf1a neo/ETn-Gm13336-Ptf1a neo mice. Bars: 200 µm. (PDF) [file pgen.1003204.s005.pdf]

Figure S6

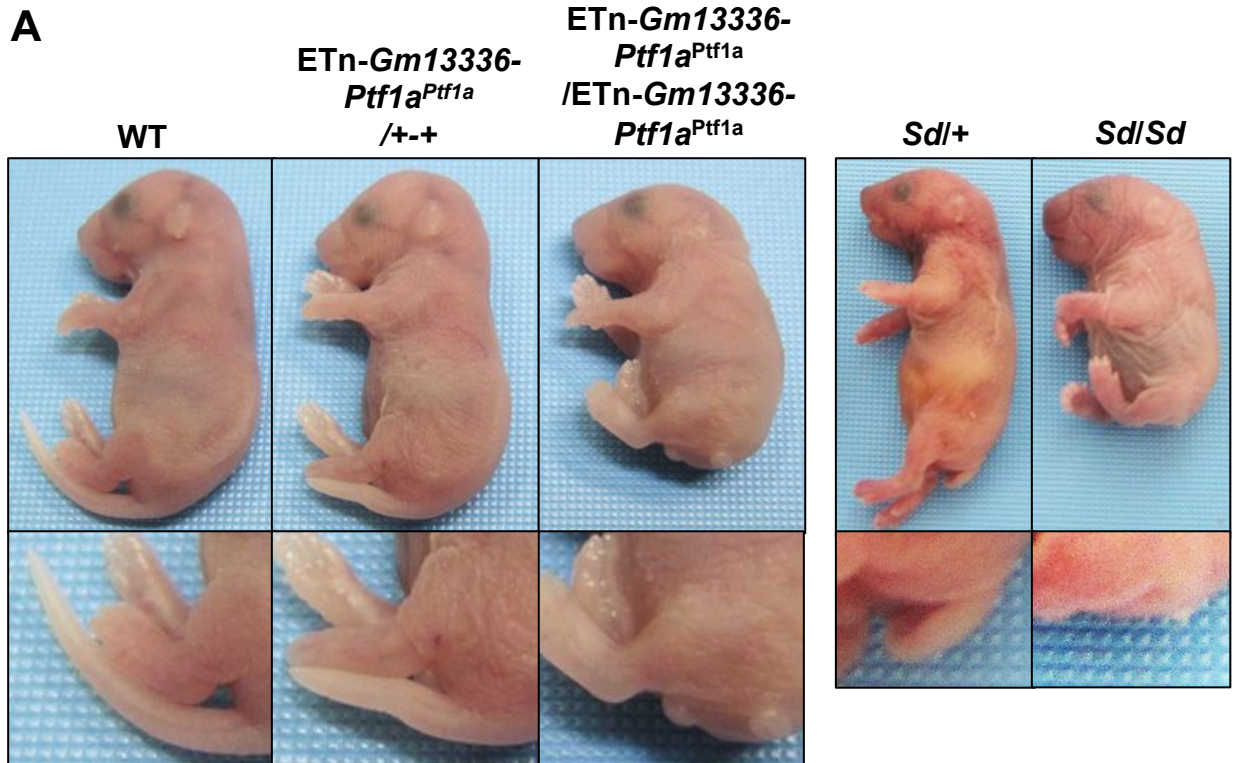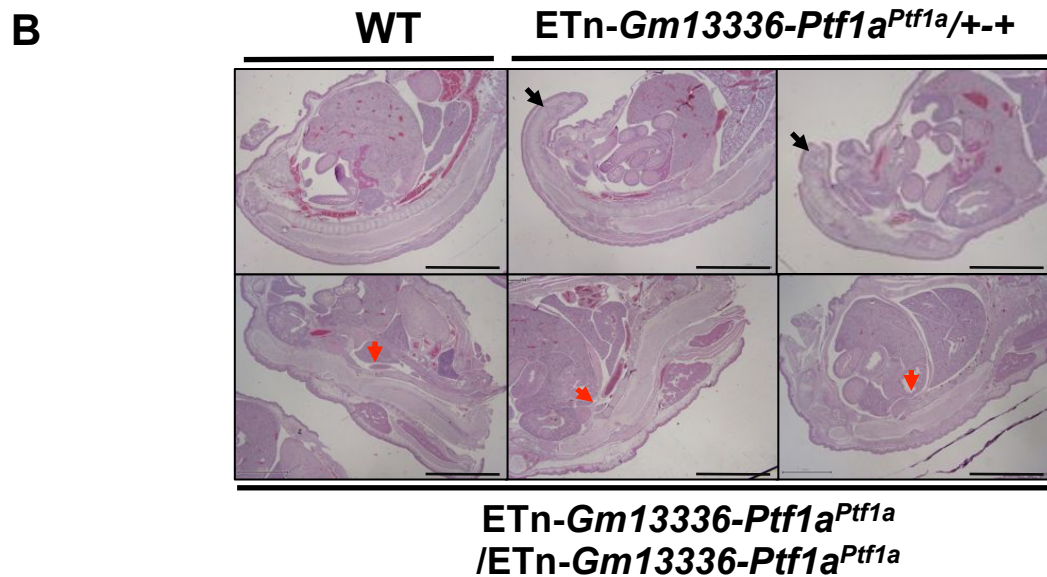

Supplement: Figure S6 — Morphology of tail of ETn-Gm13336/Ptf1a Ptf1a neonates. A. The ETn-Gm13336-Ptf1a Ptf1a/+-+ neonates showed a short tail similar to that of Sd heterozygotes, while the ETn-Gm13336-Ptf1a Ptf1a/ETn-Gm13336-Ptf1a Ptf1a neonates showed no tail and a short trunk. B. Histological examination revealed that the vertebral columns of ETn-Gm13336-Ptf1a Ptf1a/+-+ and ETn-Gm13336-Ptf1a Ptf1a/ETn-Gm13336-Ptf1a Ptf1a neonates were truncated at the eighth caudal (black arrows) and the tenth thoracic (red arrows) vertebrae, respectively. Black arrows and red arrows indicate the level of the terminal vertebral body for heterozygotes and homozygotes, respectively. Bars: 2 mm. (PDF) [file pgen.1003204.s006.pdf]

Figure S7

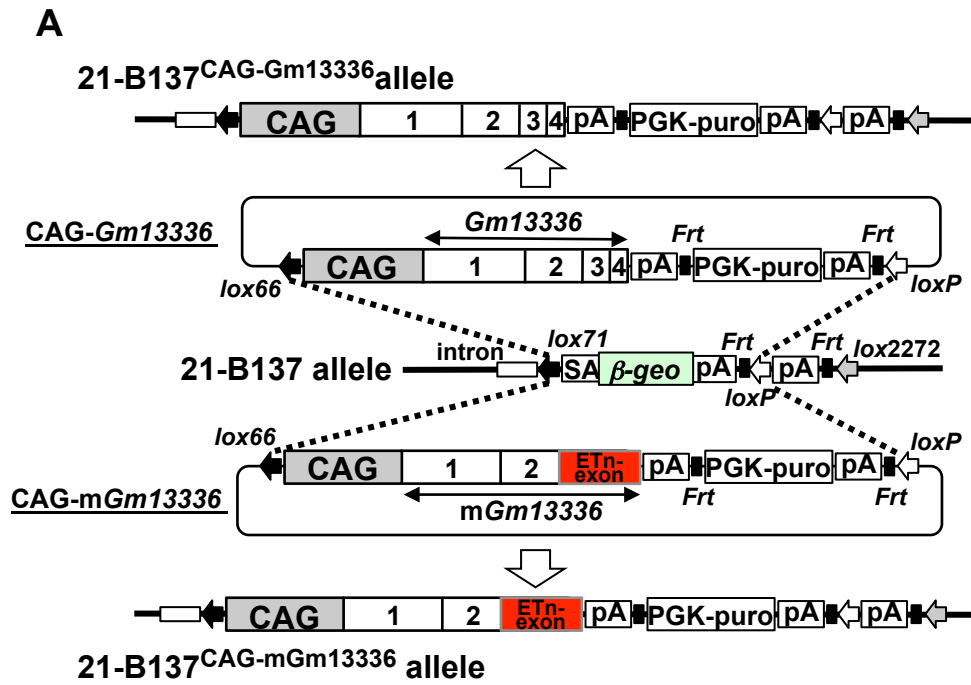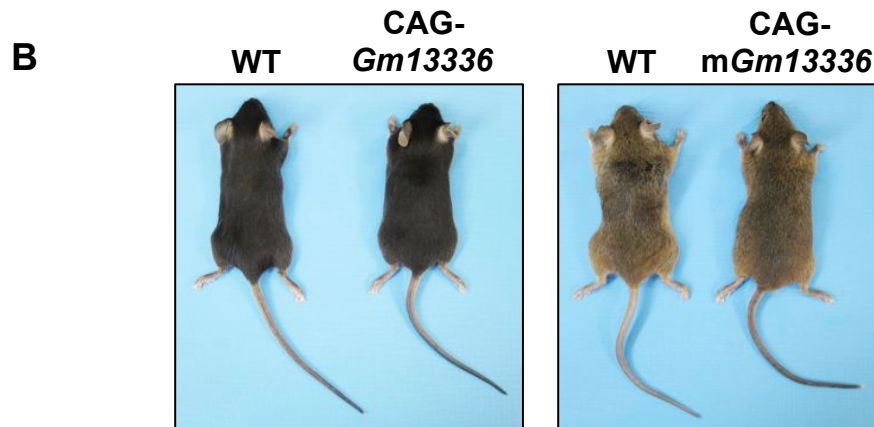

Supplement: Figure S7 — Generation and tail morphology of Gm13336-mutant mice. A. Strategy for insertion of the Gm13336 and mutant (m) Gm13336 gene into the 21-B137 locus. Normal Gm13336 cDNA and mGm13336 cDNA driven by a CAG promoter was inserted into the 21-B137 locus using Cre-mediated recombination. B. Morphology of the tail in adult CAG-Gm13336 and CAG-mGm13336 mice. The tail phenotype was normal. (PDF) [file pgen.1003204.s007.pdf]

**Figure S8**

**A E10.0**

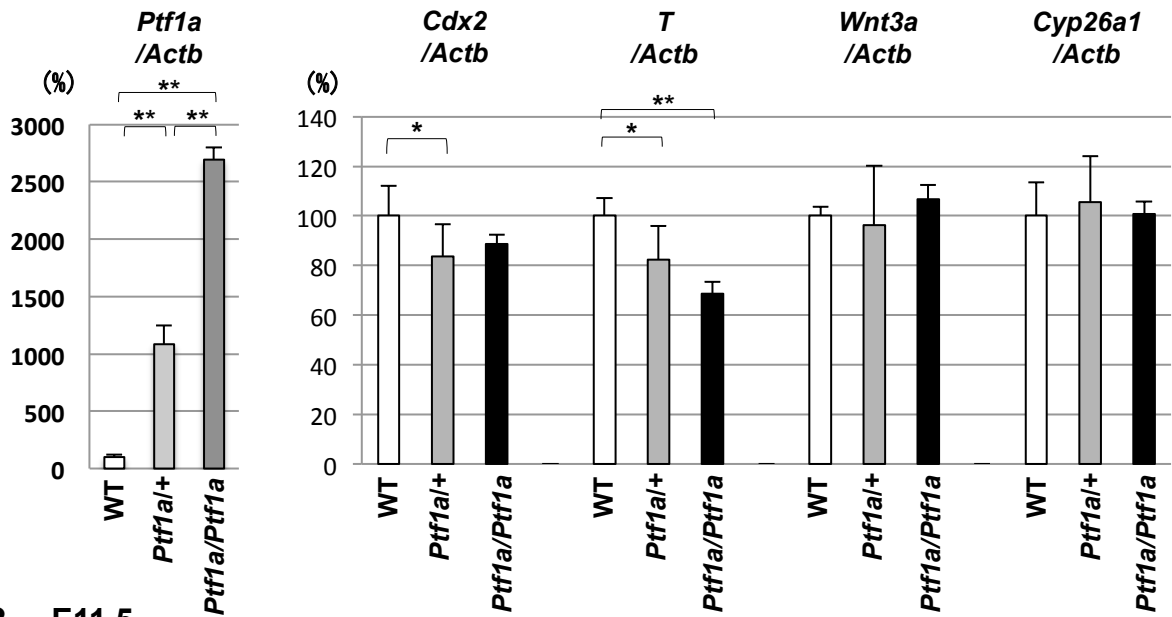

**B E11.5**

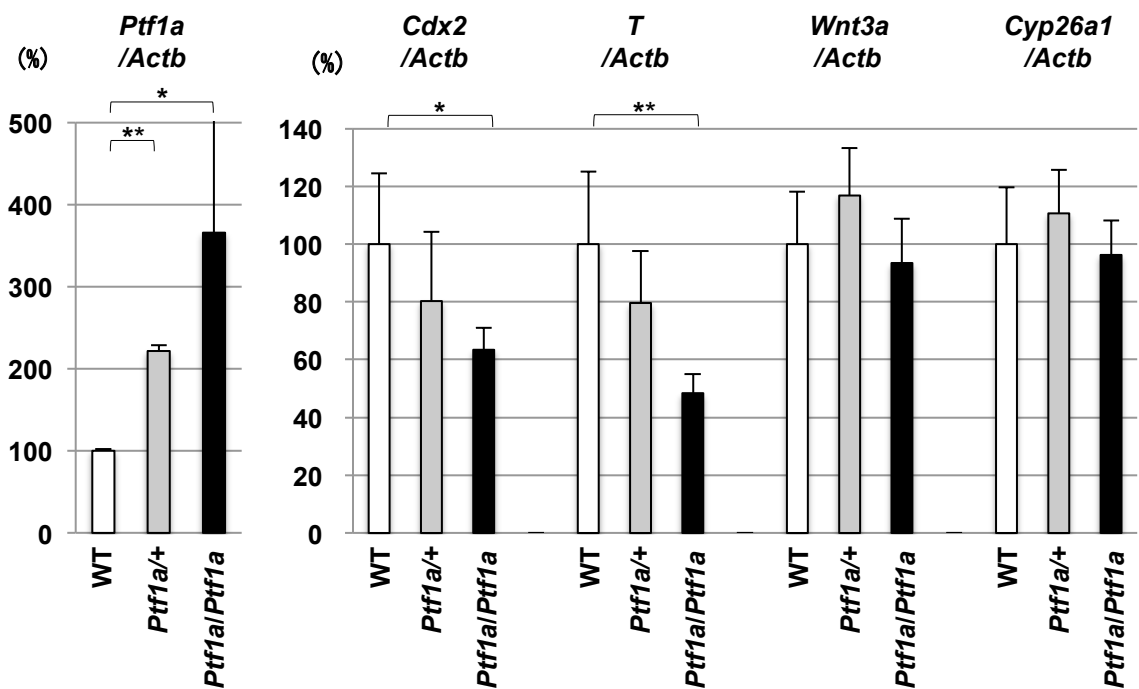

Supplement: Figure S8 — Ectopic expression of Ptf1a and downregulation of Cdx2 and its downstream targets. A. Quantitative RT-PCR analyses of the expression of Ptf1a, Cdx2, T, Wnt3a, and Cyp26a1 in the E10.0 embryos of WT, Ptf1a/+, and Ptf1a/Ptf1a littermates. Upregulation of Ptf1a and downregulation of Cdx2 and T, but not of Wnt3a and Cyp26a1 were observed. The data represent the mean ± SD of independent whole embryos (+/+: n = 4, Ptf1a/+: n = 6, Ptf1a/Ptf1a: n = 3). *p<0.05; **p<0.01. B. Quantitative RT-PCR analyses of the expression of Ptf1a, Cdx2, T, Wnt3a, and Cyp26a1 in E11.5 ETn-Gm13336/Ptf1a Ptf1a embryos of WT, Ptf1a/+, and Ptf1a/Ptf1a littermates. Upregulation of Ptf1a and downregulation of Cdx2 and T were observed. The data represent the mean ± SD of three independent whole embryos. *p<0.05; **p<0.01. (PDF) [file pgen.1003204.s008.pdf]

**Figure S9**

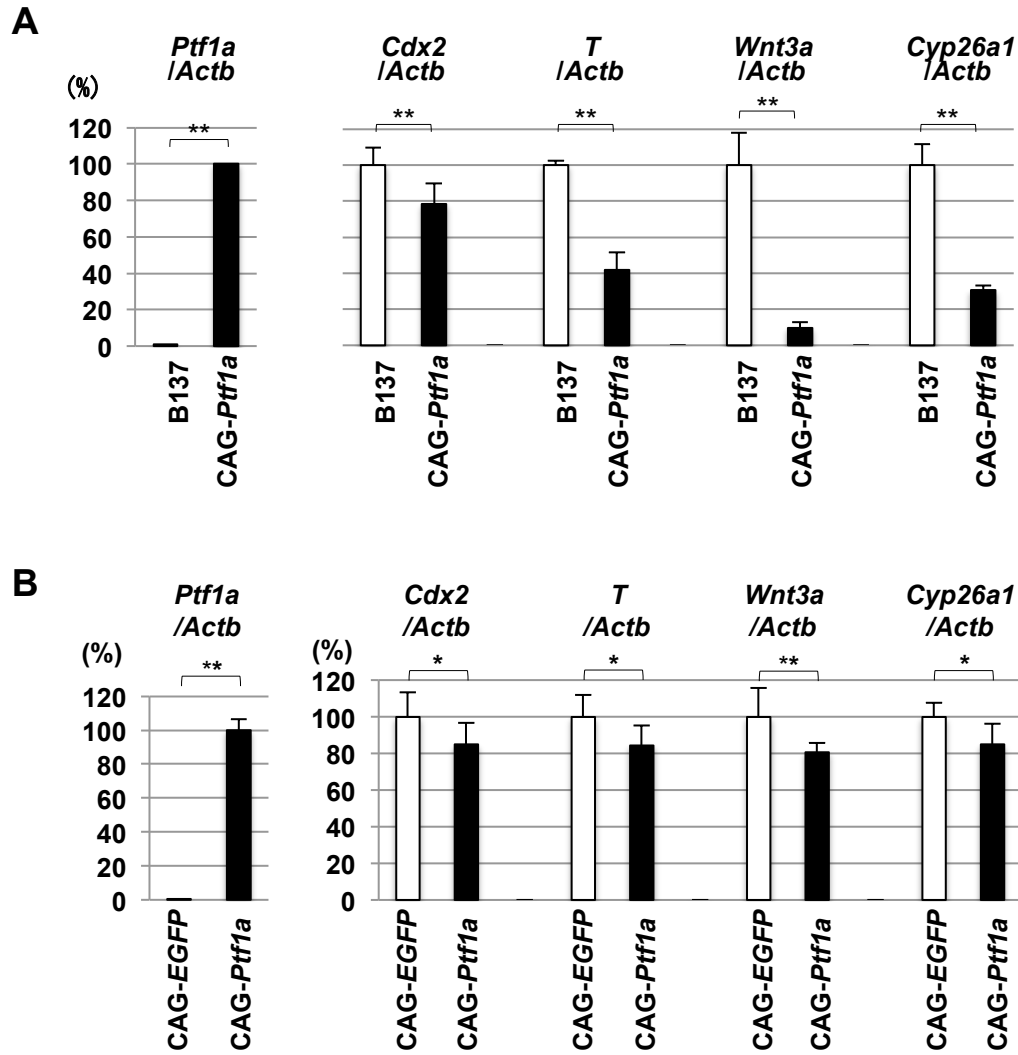

Supplement: Figure S9 — Overexpression of Ptf1a attenuates the expression of Cdx2 and its downstream targets. A. Quantitative RT-PCR analyses in ES cells with stable expression of Ptf1a. Expression of Cdx2, T, Wnt3a, and Cyp26a1 was suppressed by stable overexpression of Ptf1a. The data represent the mean ± SD of independent cultures (B137: n = 4, CAG-Ptf1a: n = 6). **p<0.01. B. Quantitative RT-PCR analyses in ES cells transfected with a CAG-EGFP expression vector (white bars) or a CAG-Ptf1a expression vector (black bars). Expression of Cdx2, T, Wnt3a, and Cyp26a1 was suppressed by transient overexpression of Ptf1a. The data represent the means ± SD of six independent cultures. *p<0.05; **p<0.01. (PDF) [file pgen.1003204.s009.pdf]
