# Supplementary material for: Ectopic Expression of Ptf1a Induces Spinal Defects, Urogenital Defects, and Anorectal Malformations in Danforth's Short Tail Mice
Source: PLoS Genet. 2013 Feb 21;9(2):e1003204. doi: 10.1371/journal.pgen.1003204 (PMC3578775; doi:10.1371/journal.pgen.1003204)

Figure S2

A

Cosmid No.	Size (bp)
C1	38kb
C2	38kb
C3	36,462
C4	37,018
C5	38,028
C6	29,398
C7	33,279
C8	35,433
C9	33,632
C10	34,497
C11	37,880
C12	37,078
C13	78,421
C14	38kb
C15	38kb
C16	35,772
C17	34,713
C18	34,719
C19	35kb

PCR fragment No.	Size (bp)
P-A1	5,033
P-A2	5,166
P-A3	5,173
P-A4	4,607
P-A5	7,371
P-A6	3,726
P-A7	5,116
P-A8	5,837
P-B1	3,902
P-B2	3,497
P-C1	6,846
P-D1	6,203
P-D2	7,298
P-E1	5,401
P-E2	4,755
P-E3	5,565
P-E4	4,901
P-E5	5,347
P-E5	5,361
P-E7	5,057
P-E8	5,032
P-E9	5,087
P-E10	2,836
P-F1	8,444
P-F2	2,509
P-F3	5,027

B

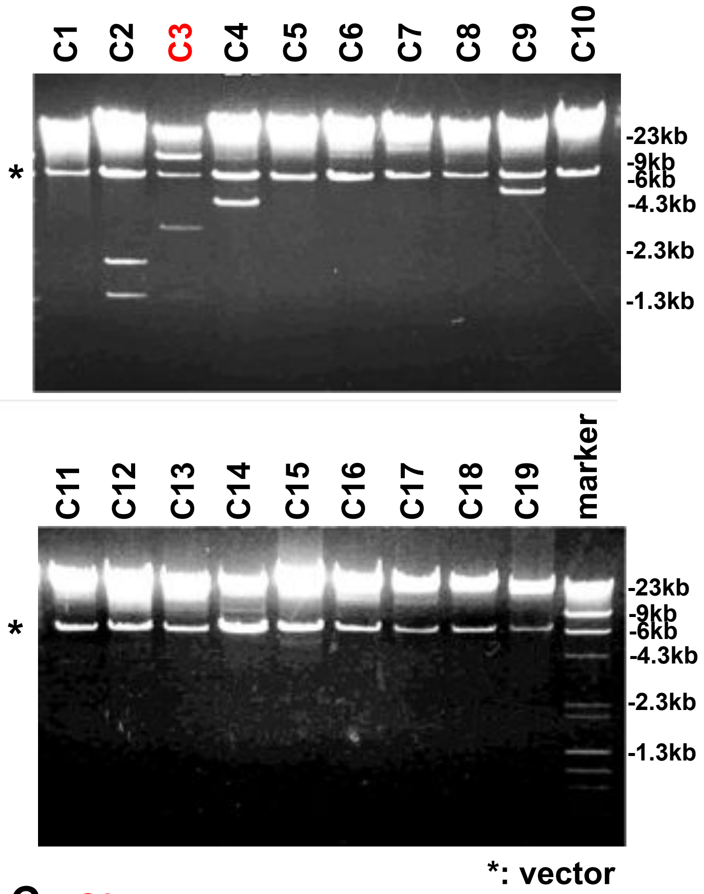

C

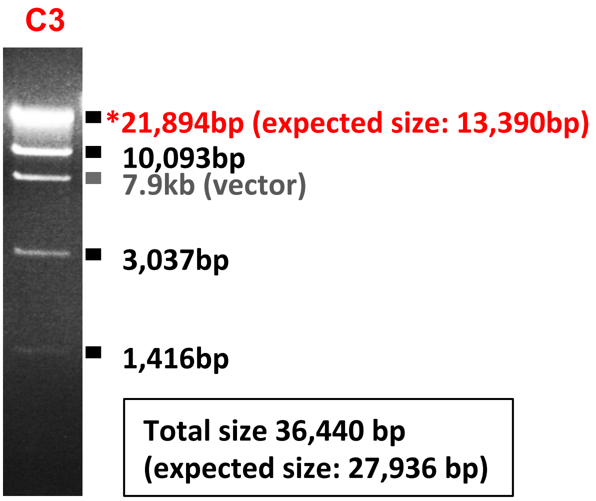

Supplement: Figure S2 — Details of cosmid clones and PCR products. A. Size of cosmid inserts and PCR products. The cosmid C3, shown in red, contains the ETn. B. DNA electrophoresis (Not I digestion) to measure the size of the cosmid clone inserts. The insert of cosmid clone C3 (shown in red) was bigger than the expected size based on its end-sequence tags and wild-type genome informatics. C. DNA electrophoresis (Not I-digestion) of the C3 cosmid clone only. This clone gave bands of 21,894-bp, 10,093-bp, 3,037-bp, and 1,416-bp (the precise band sizes were obtained after subsequent shotgun sequencing). Its total insert size (36,440-bp) was bigger than the expected size (27,936-bp). This was because of the difference in size of the largest band: 21,894-bp in the Sd-derived cosmid C3 and 13,390-bp in the wild type C57BL/6. (PDF) [file pgen.1003204.s002.pdf]
