# Supplementary material for: Ectopic Expression of Ptf1a Induces Spinal Defects, Urogenital Defects, and Anorectal Malformations in Danforth's Short Tail Mice
Source: PLoS Genet. 2013 Feb 21;9(2):e1003204. doi: 10.1371/journal.pgen.1003204 (PMC3578775; doi:10.1371/journal.pgen.1003204)
Supplement: Table S1 — Genes upregulated more than 1.7-fold in homozygous Sd embryos at embryonic day 10.0. (PDF) [file pgen.1003204.s010.pdf]

Table S1. Genes upregulated more than 1.7-fold in homozygous *Sd* embryos at embryonic day 10.0

wt_homo_log2	Gene Symbol	Gene Name
-2.292604532	*Wfdc3*	WAP four-disulfide core domain 3
-2.068914981	*Cpa1*	carboxypeptidase A1
-1.886434916	*Ptf1a*	pancreas specific transcription factor, 1a
-1.726093968	*Unc13c*	unc-13 homolog C (C. elegans)
-1.689349507	*Fancg*	Fanconi anemia, complementation group G
-1.568837495	*Saps2*	SAPS domain family, member 2
-1.50710034	*Nhsl1*	NHS-like 1
-1.450641448	*Gp1bb*	glycoprotein Ib, beta polypeptide"
-1.388891015	*Rph3al*	rabphilin 3A-like (without C2 domains)
-1.325300288	*Pramef12*	PRAME family member 12
-1.29461986	*Frmd4a*	FERM domain containing 4A
-1.223918877	*Car7*	carbonic anhydrase 7
-1.221578043	*Ikzf2*	IKAROS family zinc finger 2
-1.125159242	*Nr1d1*	nuclear receptor subfamily 1, group D, member 1
-1.107857958	*4930539N22Rik*	RIKEN cDNA 4930539N22 gene
-1.075506995	*A530054K11Rik*	RIKEN cDNA A530054K11 gene
-1.063980849	*Ugt2b34*	UDP glucuronosyltransferase 2 family, polypeptide B34"
-1.049819633	*Cyp3a11*	cytochrome P450, family 3, subfamily a, polypeptide 11"
-1.034717782	*Kirrel2*	kin of IRRE like 2 (Drosophila)
-1.027210927	*Slc38a9*	solute carrier family 38, member 9
-1.022209956	*Luc7l2*	LUC7-like 2 (S. cerevisiae)
-1.011919634	*Abcb1b*	ATP-binding cassette, sub-family B (MDR/TAP), member 1B"
-1.011841065	*Lhx1*	LIM homeobox protein 1
-0.964093392	*Sipa1l1*	signal-induced proliferation-associated 1 like 1
-0.943349826	*Slc6a16*	solute carrier family 6, member 16
-0.942660843	*Mgam*	maltase-glucoamylase
-0.940246568	*N4bp2l2*	NEDD4 binding protein 2-like 2
-0.924019703	*Nsun6*	NOL1/NOP2/Sun domain family member 6
-0.92029978	*3830417A13Rik*	RIKEN cDNA 3830417A13 gene
-0.917571744	*Ank2*	ankyrin 2, brain"
-0.898814256	*1600015I10Rik*	RIKEN cDNA 1600015I10 gene
-0.890764689	*LOC639910*	hypothetical protein LOC639910
-0.889616747	*Madcam1*	mucosal vascular addressin cell adhesion molecule 1
-0.887704018	*Igsf11*	immunoglobulin superfamily, member 11
-0.885761887	*Sectm1b*	secreted and transmembrane 1B
-0.881250083	*Olfr870*	olfactory receptor 870
-0.865447187	*6530418L21Rik*	RIKEN cDNA 6530418L21 gene
-0.864747648	*Mbnl2*	muscleblind-like 2
-0.864663626	*Cnksr2*	connector enhancer of kinase suppressor of Ras 2
-0.86407026	*5730409L17Rik*	RIKEN cDNA 5730409L17 gene
-0.862564645	*Olfr1441*	olfactory receptor 1441
-0.856683291	*Acot4*	acyl-CoA thioesterase 4
-0.854986982	*Rbpjl*	recombination signal binding protein for immunoglobulin kappa J region-like
-0.848077744	*Slc35d3*	solute carrier family 35, member D3"
-0.84462546	*Upk1a*	uroplakin 1A
-0.84351152	*Anxa7*	annexin A7
-0.842885702	*Hrg*	histidine-rich glycoprotein
-0.827769998	*Ly6g5c*	lymphocyte antigen 6 complex, locus G5C"
-0.826120079	*Gpr152*	G protein-coupled receptor 152
-0.822110043	*9130022E09*	hypothetical 9130022E09
-0.820070174	*Taar4*	trace amine-associated receptor 4
-0.807713561	*A230072E10Rik*	RIKEN cDNA A230072E10 gene
-0.796918159	*Lhx9*	LIM homeobox protein 9
-0.795244306	*Cybrd1*	cytochrome b reductase 1
-0.79479939	*Olfr1419*	olfactory receptor 1419
-0.789249795	*Veph1*	ventricular zone expressed PH domain homolog 1 (zebrafish)
-0.787280139	*Fv1*	Friend virus susceptibility 1
-0.782511125	*Taok2*	TAO kinase 2
-0.781628273	*Tgm5*	transglutaminase 5
-0.77680897	*RP23-212C14.7*	keratin associated protein 9 family member
-0.776441136	*Prm2*	protamine 2
-0.771651592	*Cdcp1*	CUB domain containing protein 1
-0.768768275	*Adrb1*	adrenergic receptor, beta 1"
-0.76817615	*Tmem89*	transmembrane protein 89
-0.767808688	*Sfxn3*	sideroflexin 3
-0.766146098	*Rhox6*	reproductive homeobox 6
