# Supplementary material for: Ectopic Expression of Ptf1a Induces Spinal Defects, Urogenital Defects, and Anorectal Malformations in Danforth's Short Tail Mice
Source: PLoS Genet. 2013 Feb 21;9(2):e1003204. doi: 10.1371/journal.pgen.1003204 (PMC3578775; doi:10.1371/journal.pgen.1003204)
Supplement: Table S2 — Genes downregulated more than 1.7-fold in homozygous Sd embryos at embryonic day 10.0. (PDF) [file pgen.1003204.s011.pdf]

Table S2. Genes downregulated more than 1.7-fold in homozygous *Sd* embryos at embryonic day 10.0

wt_homo_log2	GeneSymbol	GeneName
0.766372156	*Ccdc88a*	coiled coil domain containing 88A
0.771837649	*Myf6*	myogenic factor 6
0.772864656	*Dgkd*	diacylglycerol kinase, delta
0.774188515	*Rora*	RAR-related orphan receptor alpha
0.779194956	*Ccdc148*	coiled-coil domain containing 148
0.792052297	*Erdr1*	erythroid differentiation regulator 1
0.793156058	*LOC100047943*	similar to Ina protein
0.80952905	*Rabggtb*	RAB geranylgeranyl transferase, b subunit"
0.812644809	*Gpx6*	glutathione peroxidase 6
0.814251994	*2310035P21Rik*	RIKEN cDNA 2310035P21 gene
0.833473988	*Fabp1*	fatty acid binding protein 1, liver
0.842128041	*Rian*	RNA imprinted and accumulated in nucleus
0.851771248	*Tigd3*	tigger transposable element derived 3
0.887936059	*Nkx1-2*	NK1 transcription factor related, locus 2 (Drosophila)"
0.896362994	*Mid1*	midline 1
0.908795458	*Arv1*	ARV1 homolog (yeast)
0.912931803	*EG633640*	predicted gene, EG633640
0.957347792	*Aldh1a3*	aldehyde dehydrogenase family 1, subfamily A3"
1.016807594	*Fzd2*	frizzled homolog 2 (Drosophila)
1.024433763	*Lrtm1*	leucine-rich repeats and transmembrane domains 1
1.035904587	*1300002K09Rik*	RIKEN cDNA 1300002K09 gene
1.097376463	*Slc15a2*	solute carrier family 15 (H+/peptide transporter), member 2
1.134145103	*Abcb10*	ATP-binding cassette, sub-family B (MDR/TAP), member 10
1.288954763	*Ccdc151*	coiled-coil domain containing 151
1.346441658	*Car3*	carbonic anhydrase 3
1.462164049	*Cdx2*	caudal type homeo box 2
1.606081542	*Pard3*	par-3 (partitioning defective 3) homolog (C. elegans)
1.662669172	*Nkx3-1*	NK-3 transcription factor, locus 1 (Drosophila)"
1.981420785	*T*	brachyury
2.011016198	*Plp1*	proteolipid protein (myelin) 1
2.107707874	*4921530L18Rik*	RIKEN cDNA 4921530L18 gene
2.19894211	*Mid1*	midline 1
3.017687813	*Tnfrsf14*	tumor necrosis factor receptor superfamily, member 14 (herpesvirus entry mediator)
