# Supplementary material for: Ectopic Expression of Ptf1a Induces Spinal Defects, Urogenital Defects, and Anorectal Malformations in Danforth's Short Tail Mice
Source: PLoS Genet. 2013 Feb 21;9(2):e1003204. doi: 10.1371/journal.pgen.1003204 (PMC3578775; doi:10.1371/journal.pgen.1003204)
Supplement: Text S1 — Supplementary Materials and Methods. Cosmid library of Sd homozygotes, DNA sequencing, Extraction and reverse transcription of RNA, Cloning of the Gm13336 cDNA, Skeletal preparations, X-ray computed tomography, Establishment of an Sd/+ ES cell line, Construction of replacement vectors for CAG-Gm13336 and CAG-mGm13336, and establishment of Ayu21-B137CAG-Gm13336 and Ayu21-B137CAG-mGm13336 mouse lines, Transfection of CAG-Ptf1a or CAG-EGFP expression vectors into ES cells, Microarray analysis methods are provided. (DOCX) [file pgen.1003204.s012.docx]

**Text S1 for**

**Ectopic Expression of *Ptf1a* Induces Spinal Defects, Urogenital Defects, and Anorectal Malformations in *Danforth’s Short Tail* Mice**

**Cosmid library of *Sd* homozygotes**

As a step in the positional cloning of the *Sd* gene, we created a physical map of the *Sd* candidate region. A mouse genomic cosmid library derived from embryonic fibroblast cells of homozygous *Sd* mutants was constructed using the methods described in the Instruction Manual of the SuperCos1 Cosmid Vector Kit (Stratagene, La Jolla, CA). High-molecular-weight genomic DNA prepared from embryonic fibroblast cells of embryonic day (E) 11.5 homozygous *Sd* embryos was partially digested with Bam HI restriction endonuclease and size-fractionated by pulsed-field gel electrophoresis. E11.5 homozygous *Sd* embryos were generated by intercrossing heterozygous [*Sd +/+* *Skt^Gt^*; *trans* configuration] mice with a C57BL/6 genetic background. *Skt^Gt^* provides a marker to genotype embryos for the *Sd* mutation. The partially digested DNA was then ligated to Bam HI-digested and dephosphorylated pSuperCos1 cosmid vector and introduced into competent cells using a Gigapack® III gold packaging extract (Stratagene). To create a cosmid contig of the *Sd* region, the genomic cosmid library was screened with thirty-two digoxigenin-labeled probes generated from four genes—*Ptf1a*, 4921504E06Rik, *Otud1*, and *Skt*—and unique genomic sequences in the *Sd* locus. Based on physical mapping of 19 cosmids and 25 PCR products, the assembled contig spans a 542-kb region on mouse chromosome 2 that contains the *Sd* locus.

**DNA sequencing**

Four cosmid clones spanning the *Sd* region were sequenced by the shotgun method. Briefly, the clones were fragmented by sonication, then DNA fragments of 1.5–6.0-kb were subcloned into pBluescriptII SK(–). Four hundred randomly selected clones were sequenced at both ends with T3 and T7 dye terminators using a capillary-based autosequencer (ABI 3700; PE Applied Biosystems, Foster City, CA). DNA sequences were assembled using PolyPhred software [1]. Gaps between the assembled segments were connected by direct cosmid sequencing using primers designed from the end sequences of the assembled segments; we thus obtained a genomic DNA sequence comprising 36,440 nucleotides from the cosmid clone whose insert size was bigger than that predicted by wild-type genome informatics.

**Extraction and reverse transcription of RNA**

Total RNA was extracted and purified using an RNeasy mini kit (Qiagen, Valencia, CA) in combination with DNA digestion using DNase (Qiagen). Purified RNA was reverse-transcribed using the Thermoscript RT-PCR system (Invitrogen, Carlsbad, CA). All procedures were performed according to the manufacturer’s protocols.

**Cloning of the *Gm13336* cDNA**

The full-length cDNA of *Gm13336* were isolated by rapid amplification of cDNA ends (RACE) using 5′ RACE and 3′ RACE systems (Invitrogen) according to the manufacturer’s instructions. Total RNA from E9.5 *Sd*/*Sd* embryos, *Sd*/+ embryoid bodies, and CAG-*Gm13336* embryonic stem ES cells was extracted using an RNeasy mini kit (Qiagen). First-strand cDNA synthesis from 500 ng of total RNA was performed using a Thermoscript RT-PCR system (Invitrogen). PCRs to amplify the full-length normal and mutant *Gm13336* gene were performed using gene-specific primer pairs. The initial PCR for 5′ RACE was performed using the primer AK-5′RACE-A1 (5′-CTTCCCATCCCCTTACTTTG-3′) in the *Gm13336* sequence and the anchor primer (5′-GGCCACGCGTCGACTAGTACGGGiiGGGiiGGGiiG-3′) (Invitrogen). The nested PCR was performed using the primer AK-5′RACE-A2 (5′-AGTCCATCAACGACGCCTTC-3′) in the *Gm13336* sequence and the amplification primer (5′-GGCCACGCGTCGACTAGTAC-3′) in the anchor primer sequence. The initial PCR for 3′ RACE was performed using the primer AK-3′RACE-S1 (5′-CAAAGTAAGGGGATGGGAAG-3′) in the *Gm13336* sequence and the amplification primer (5′-GGCCACGCGTCGACTAGTAC-3′) (Invitrogen). The nested PCR for 3′ RACE was performed using primer AK-3′RACE-S2 (5′-GTGACGCTTTGTGAGTGATCCGTGGC-3′) in the *Gm13336* sequence and the amplification primer (5′-GGCCACGCGTCGACTAGTAC-3′) in the anchor primer sequence. The PCR conditions for both reactions were 25 cycles of 94°C for 45 s, 57°C for 45 s, and 72°C for 1 min, using 0.5 units of LA Taq polymerase (Takara, Otsu, Japan). Amplified fragments were sequenced directly by Big Dye Terminator Cycle Sequencing (PE Applied Biosystems).

**Skeletal preparations**

The skin was peeled off the embryos, then they were fixed in 95% ethanol for 3 days. Embryos were cleared by placing them in 1% KOH for 1 day after staining by alcian blue and alizarin red. Excess stain was removed with 2% KOH, then the samples were transferred to glycerol [2].

**X-ray computed tomography**

The morphology of the dens and sacrum of *Sd* heterozygous mutant mice was imaged at a pixel size of 9 µm using SkyScan 1076; this facilitated volumetric reconstruction for two- and three-dimensional quantitative analysis and realistic three-dimensional visualization by SkyScan software.

**Establishment of an *Sd*/+ ES cell line**

ES cells were cultured at 37°C in a humidified atmosphere of 6.5% CO_2_ in air. Blastocysts were plated individually into a 48-well plate coated with 0.15% gelatin in KSR-GMEM medium. This medium consists of Glasgow Minimum Essential Medium (Sigma, St. Louis, MO) with 1 × nonessential amino acids (Invitrogen), 0.1 mM β-mercaptoethanol, 1 mM sodium pyruvate, 1% fetal bovine serum (HyClone; Thermo Fisher Scientific Inc., UT, USA), 14% Knockout™ Serum Replacement (Invitrogen), and 1100 U/ml leukemia inhibitory factor (ESGRO; Chemicon, Temecula, CA). The blastocysts were allowed to hatch and attach to the dish and were fed every 3 days with KSR-GMEM medium. After 10 days in culture, the inner cell mass outgrowth was dissociated in threefold-diluted 0.25% trypsin/1 mM EDTA (Sigma), and then plated onto a 24-well plate with a feeder layer of mitomycin C-treated primary mouse embryo fibroblasts. After this first passage, the ES cells were gradually plated onto larger culture plates with feeder layers. ES cells were routinely passaged and diluted five- to six-fold every 2 days, and the medium was changed on alternate days. To establish germline-competent *Sd*/+ ES cell lines, we successfully obtained and stocked 15 ICM-derived colonies from an *Sd*/+ male with a C57BL/6 background crossed with a CBA female. The sex of the established ES cell lines was examined by genomic PCR to detect the *Sry* locus on the Y chromosome; six lines were *Sry*-positive, meaning that they were male ES cell lines. To detect the *Sry* locus, the 5′ and 3′ primers Sry-F (5′-TGACTGGGATGCAGTAGTTC-3′) and Sry-R (5′-TGTGCTAGAGAGAAACCCTG-3′), located in the *Sry* locus, generated a 240-bp fragment from ES cell lines carrying the Y chromosome. The six established male ES cell lines were also examined by genomic PCR to detect the ETn in the *Sd* locus, and three of the six lines were both *Sry*-positive and ETn-positive, meaning that they were male *Sd*/+ ES cell lines. To compare chimera production efficiency, the three ES cell lines were aggregated with ICR morulas. Two ES cell lines resulted in production of male chimeric mice with 100% contribution of ES cells, as shown by coat color, and showed a heterozygous *Sd* phenotype. All of the complete chimeras were able to pass the ES cell genome onto the next generation.

**Construction of replacement vectors for CAG-*Gm13336* and CAG-m*Gm13336*, and establishment of Ayu21-B137^CAG-Gm13336^ and Ayu21-B137 ^CAG-mGm13336^ mouse lines**

The 917-bp and 1,105-bp normal and mutant (m)*Gm13336* cDNA fragments, respectively, were cloned into the pGEM-T Easy Vector (Promega, Madison, WI). These clones were used to produce replacement vectors that contained *lox66*, CAG-*Gm13336* or CAG-m*Gm13336* cDNA, a polyadenylation site, PGK-puro, another polyadenylation site, and *loxP*. The *β-geo* gene in Ayu21-B137 ES cells was replaced with the replacement vector by Cre-mediated recombination to establish CAG-*Gm13336* and CAG-m*Gm13336* mouse lines. The Ayu21-B137 clone (http://egtc.jp/action/access/clone_detail?id=21-B137) was chosen because Ayu21-B137 heterozygous and homozygous animals appear normal and are fertile. ETn-*Gm13336*/*Ptf1a*^CAG-^*^Gm13336^* mice and ETn-*Gm13336*/*Ptf1a*^CAG-m^*^Gm13336^* mice were backcrossed to C57BL/6 mice for at least five generations.

**Transfection of CAG-*Ptf1a* or CAG-*EGFP* expression vectors into ES cells**

ES cells were cultured in KSR-GMEM (as described above) containing 1100 U/ml leukemia inhibitory factor (ESGRO; Chemicon, Temecula, CA). The Nucleofection® System (Lonza Cologne GmbH; Köln, Germany) was used for DNA electroporation to introduce the CAG-*Ptf1a* or CAG-*EGFP* expression vector into ES cells. Prior to electroporation, cultured mouse ES cells were washed twice with phosphate-buffered saline and detached from the substrate by five minutes of incubation with 0.025% trypsin-EDTA at 37°C. The trypsin was neutralized by incubating the cells in medium in which the KSR in KSR-GMEM was replaced with a final concentration of 15% fetal bovine serum. The detached cells were then dissociated by the addition of 8 ml/dish of medium followed by gentle pipetting. The cells were pelleted by centrifugation at 90 × *g* for 3 min and resuspended at a density of 10 × 10^6^ cells/ml in Nucleofector^™^ solution with the supplied supplement added (Lonza). For each electroporation, 100 µl of Nucleofector solution with 50 μg of the plasmid vector pCAG-*Ptf1a* or pmaxGFP™ (Lonza) were placed in a cuvette (Lonza), and were electroporated using the supplied setting for mouse embryonic stem cells. After electroporation, the contents of each cuvette were dispersed as rapidly as possible with 8 ml of KSR-GMEM medium, and then transferred to a 10-cm dish. mRNA was harvested from these ES cells 24 h after transfection.

**Microarray analysis**

A Whole Mouse Genome Array system ver.2.0 (Agilent Technologies, Santa Clara, CA) was used in this study. Total RNA, isolated using an RNeasy mini kit (Qiagen) in combination with DNA digestion using DNase (Qiagen), was hybridized to the slides. Hybridized slides were washed and scanned using a microarray scanner (Agilent Technologies). Data were analyzed with the Feature Extraction software ver. 10.5 (Agilent Technologies).

**References**

1. Nickerson DA, Tobe VO, Taylor SL (1997) PolyPhred: automating the detection and genotyping of single nucleotide substitutions using fluorescence-based resequencing. *Nucleic Acids Res* 25:2745–2751.

2. Hogan B, Beddington R, Costantini F, Lacy E (1994) Manipulating the Mouse Embryo. A laboratory manual, Cold Spring Harbor Laboratory Press.
